# Sequential Application of Ligand and Structure Based Modeling Approaches to Index Chemicals for Their hH_4_R Antagonism

**DOI:** 10.1371/journal.pone.0109340

**Published:** 2014-10-16

**Authors:** Matteo Pappalardo, Nir Shachaf, Livia Basile, Danilo Milardi, Mouhammed Zeidan, Jamal Raiyn, Salvatore Guccione, Anwar Rayan

**Affiliations:** 1 Department of Chemical Sciences, University of Catania, Catania, Italy; 2 Drug Discovery Informatics Lab, QRC-Qasemi Research Center, Al-Qasemi Academic College, Baka El-Garbiah, Israel; 3 Etnalead s.r.l., Scuola Superiore di Catania, University of Catania, Catania, Italy; 4 National Research Council, Institute of Biostructures and Bioimaging, Catania, Italy; 5 Department of Pharmaceutical Sciences, University of Catania, Catania, Italy; Medical School of Hannover, Germany

## Abstract

The human histamine H_4_ receptor (hH_4_R), a member of the G-protein coupled receptors (GPCR) family, is an increasingly attractive drug target. It plays a key role in many cell pathways and many hH_4_R ligands are studied for the treatment of several inflammatory, allergic and autoimmune disorders, as well as for analgesic activity. Due to the challenging difficulties in the experimental elucidation of hH_4_R structure, virtual screening campaigns are normally run on homology based models. However, a wealth of information about the chemical properties of GPCR ligands has also accumulated over the last few years and an appropriate combination of these ligand-based knowledge with structure-based molecular modeling studies emerges as a promising strategy for computer-assisted drug design. Here, two chemoinformatics techniques, the Intelligent Learning Engine (ILE) and Iterative Stochastic Elimination (ISE) approach, were used to index chemicals for their hH_4_R bioactivity. An application of the prediction model on external test set composed of more than 160 hH_4_R antagonists picked from the chEMBL database gave enrichment factor of 16.4. A virtual high throughput screening on ZINC database was carried out, picking ∼4000 chemicals highly indexed as H_4_R antagonists' candidates. Next, a series of 3D models of hH_4_R were generated by molecular modeling and molecular dynamics simulations performed in fully atomistic lipid membranes. The efficacy of the hH_4_R 3D models in discrimination between actives and non-actives were checked and the 3D model with the best performance was chosen for further docking studies performed on the focused library. The output of these docking studies was a consensus library of 11 highly active scored drug candidates. Our findings suggest that a sequential combination of ligand-based chemoinformatics approaches with structure-based ones has the potential to improve the success rate in discovering new biologically active GPCR drugs and increase the enrichment factors in a synergistic manner.

## Introduction

G-protein coupled receptors (GPCRs) are the largest integral membrane protein family in the human genome. They have a typical structural topology consisting of seven transmembrane helices (7TMH) connected by intracellular and extracellular loops, with an extracellular N-terminal and an intracellular C-terminal [1]. GPCRs derive their name from their ability to recruit and regulate the activity of intracellular heterotrimeric G-proteins. GPCRs are also known as seven-transmembrane domain (7TM), heptahelical, serpentine and G protein-linked (GPLR) receptors. Their main role is to transduce a signal across the cell membrane. GPCRs are grouped into 6 classes (A-F) based on sequence homology and functional similarity [2], [3].

The H_4_ histamine - a physiological amine that regulates the inflammatory response - receptor (H_4_R) belongs to class “A” of the GPCRs. To date, four histamine receptors are known (H_1_R, H_2_R, H_3_R and H_4_R) [4]. Human H_4_R (hH_4_R) is the most recently discovered, over a decade ago on the basis of its high sequence homology with the H_3_ receptor [5], [6], [7], [8], [9]. The discovery of this fourth histamine receptor, and the evidence that it is expressed in many cell types involved in allergic responses, suggested that hH_4_R may play an important role in chemotaxis, allergy, inflammation, autoimmune disorders and acts as a mediator release in various types of immune cells [10]. Recent studies suggest the hH_4_R as modulator in cancer, neuropathic pain, vestibular disorders and type 2 diabetes. The hH_4_R is widely distributed, especially in organs associated with the immune system [11], [12]. It is preferentially expressed in intestinal tissue, spleen, thymus, medullary cells, bone marrow and peripheral hematopoietic cells, including eosinophils, basophils, mast cells, T lymphocytes, leukocytes and dendritic cells [13], [14]. These cell types are primarily involved with the development and continuation of allergic responses. Based on experiments using animal models, hH_4_R antagonists show reasonable therapeutic potential for treatment of allergy, inflammation, asthma and colitis [15], [16], [17], [18]. Much of the recent drug research in hH_4_R field is focused on antagonists, mainly due to the prospective of new pharmacotherapies for the treatment of inflammatory diseases. hH_4_R characterization clearly indicates the potential of this receptor as a novel drug target for treating allergy and inflammation. Thus, more effective search for potent and selective hH_4_R antagonists is in progress to explore the therapeutic potential of such compounds [19]. Due to the lack of experimental 3D-structure ofhH_4_R, structure based virtual screening campaigns demand highly accurate models.

Homology modeling is by now an established method [20], [21] and is expected to be successful for modeling of the GPCR super-family. However, in their natural milieu GPCRs are embedded in a membrane environment which is not reproduced in the normally available homology modeling strategies. Up to date, the vast majority of virtual screening campaigns of hH4R ligands used homology models refined by energy minimization steps [22], [23], [24] but it is still open to debate whether molecular dynamics may significantly improve the quality of the constructed models of hH_4_R in terms of enrichment factors.

As an alternative, ligand-based *in silico* techniques (including, pharmacophore and chemo-informatic tools) are increasingly used to distinguish active from inactive chemicals and search large databases for novel bioactive products [25], [26], [27]. Chemo-informatic tools which use optimization methods such as Genetic Algorithms(GA) [28], [29], Neural Networks(NN) [30], [31], Monte Carlo(MC), Simulated Annealing(SA) [32], k-nearest neighbor (kNN) [33], [34], Support Vector Machines(SVM) [35], [36] or Bayesian Classifiers and some of their combinations(MCSA) [34], [37], [38], [39], [40], are many times considered to be more useful than Molecular Docking, which is limited to targets with known 3D structures [41]. Molecular Docking often gives unacceptable number of false positives and false negatives and is very time consuming for screening large databases [42]. It is thought that a combined approach of structure- and ligand-based virtual screening methods would increase the chance of identifying bioactive chemicals [43], [44], [45].

Recently, some general purpose optimization algorithms for screening multi dimensional spaces and detect global and local minima [46], [47] have been modified to solve chemoinformatics problems and used to index chemicals for their molecular bioactivity [48], [49], [50]. Iterative Stochastic Elimination (ISE) is an efficient method for searching a combinatorial space in order to detect the best set of solutions. Firstly it was applied for solving bioinformatics problems such as positioning protons [51], predicting side chains conformations [52] and searching conformational space of cyclic peptides [53] and loops [54]. Later it was adapted to solve other chemo-informatic problems such as selecting a certain set of descriptors out of a large set and optimizing the ranges of the descriptors to obtain the best solution for differentiating between databases. The results of the optimization of descriptors and ranges is employed for indexing chemicals according to their bioactivity and prioritizing molecules in large databases [49], [50]. The intelligent learning engine (ILE) is a system normally employed for the setting up of prediction models. Implementation of ILE enables to choose from a large number of candidates those with the largest probability to have a certain property, e.g. for a molecule to be a drug candidate for a certain disease. The logic of ILE is application-independent and can be used in a variety of fields. However, it has been applied so far to solve problem in bioinformatic and cheminformatic fields only. Intelligent Learning Engine (ILE) has been shown to construct highly efficient prediction models for molecular activity indexing [46], [48].

In this study both these tools were applied to search the ZINC database for the presence of potential hH4R ligands that were collected in a focused set of compounds which were docked to an optimized hH4R 3D model leading to a consensus library of highly promising drug candidates. The hH_4_R 3D structure was predicted by homology modeling using the crystal structure of H_1_receptor as the template, then refined by energy minimization. Next, all-atom Molecular Dynamics simulations of the membrane-embedded hH4R structure were carried out in order to equilibrate the whole system in an environment as similar as possible to its physiological milieu. The reliability of the 3D models was validated by docking studies which evaluated the ability of the different models to separate active from inactive ligands.

## Methods

**Figure 1:**
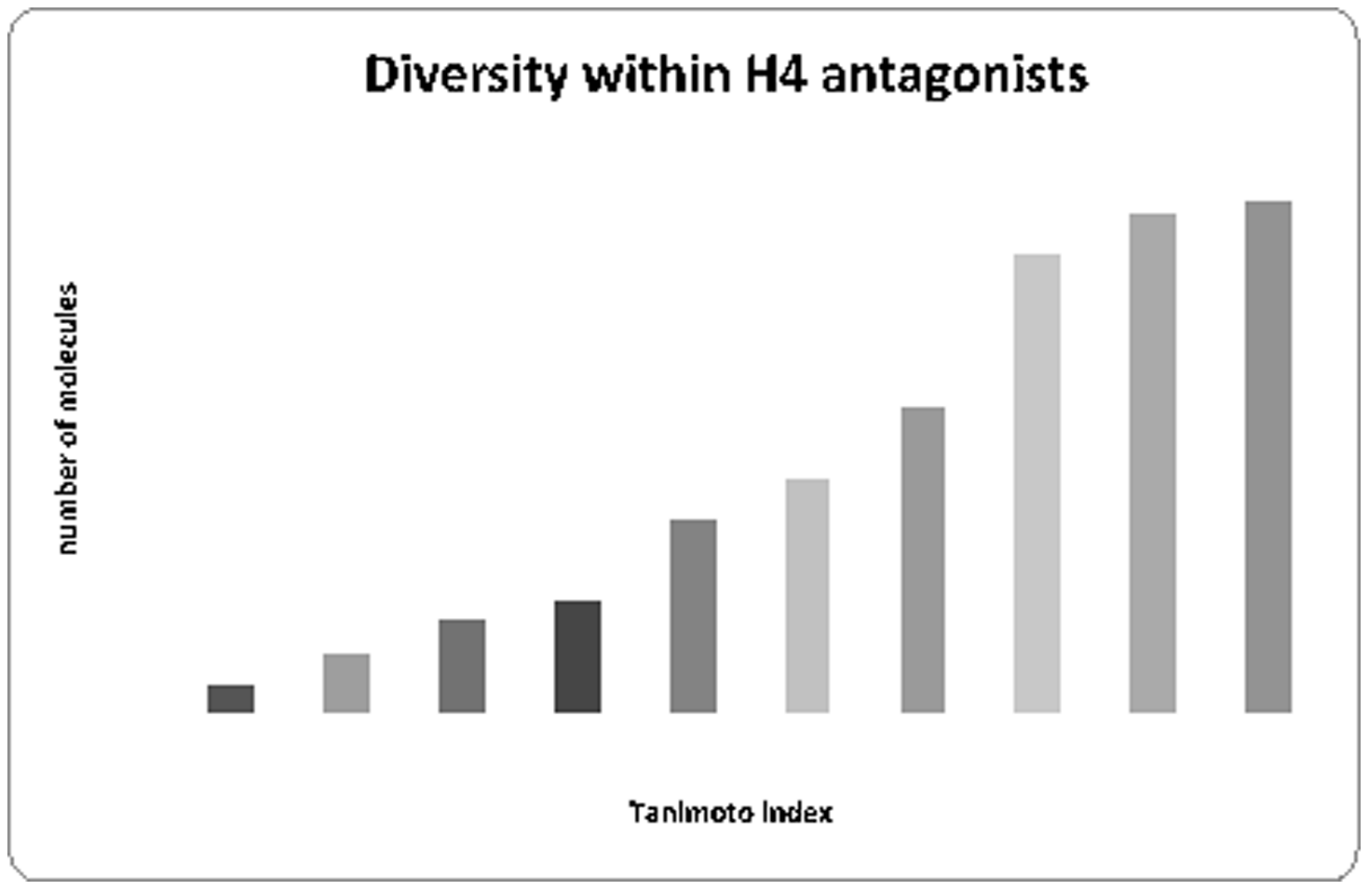
Diversity within hH_4_R antagonists that were used for modeling (training and test sets).

Literature was surveyed to collect unique 78 hH_4_R antagonists [16], [55], [56], [57], [58], [59] with IC_50_ less than 10 µM (dataset in smile format is presented in File S1). Nine thousands molecules were selected randomly from the ZINC database to represent a set of presumably inactive molecules at hH_4_R. This is justified since prediction models used for virtual screening should cover the space of properties of chemicals from the screened database. Using decoys that are similar to the active compounds will not be effective for constructing prediction models applicable for screening ZINC database with different properties' space. For similarity search we have used extended connectivity fingerprint (ECFP4) with Tanimoto similarity score. The selected molecules from ZINC are highly diverse and were examined for their structural distance from known hH_4_R antagonists (Tanimoto index <0.2). It is worth to assign that structural distance is the highest Tanimoto index in comparison to all known hH_4_R antagonists. The diversity within the hH_4_R antagonists is shown in figure 1. It is worth to assign that the diversity of learning sets could have high impact on covered properties space and may affect the quality of the obtained models of prediction. In our study, the diversity of the H4 receptor antagonists is acceptable but not high enough to ensure high quality models covering large properties space.

Two hundred thirty seven hH_4_R antagonists with IC_50_/KI less than 10 µM were extracted from chEMBL database (dataset in smile format is presented in File S2). They were compared to the 78 hH_4_R antagonists and identical ones were discarded. The remaining 160 chemicals were used as an independent external test set. Physico-chemical properties of all chemicals were calculated using MOE 2D descriptors[MOE. (The Molecular Operating Environment) Version 2009.10, Chemical Computing Group Inc., 1010 Sherbrooke Street West, Suite 910, Montreal, Canada H3A 2R7. http://www.chemcomp.com.]

The 2D descriptors are based on properties such as molecular weight, total charge and charge distributions, H-bond donors/acceptors, log P, solubility, types of atoms and so forth (http://www.chemcomp.com/journal/descr.htm).

To assess the predictability of the proposed model, the data sets of antagonists/non-actives has been split into 66.7% (52/6000 hH_4_R antagonists/non actives) for the training set and 33.3% (26/3000 hH_4_R antagonists/non actives) for the test set. The training and test sets were generated by random picking. Two ligand-based chemoinformatics techniques were utilized for virtual screening of large chemical databases: The first one is the Intelligent Learning Engine (ILE) which is a commercial software developed by RAND Biotechnologies LTD. As described by the vendors it is a prediction learning system, which enables selection from a large number of items a small number of items with the highest probability to possess a specific functionality. The input is a set of actives/non-active compounds. The chemicals are encoded as a binary vector comprising a plurality of binary descriptors. Each descriptor characterizes a certain property of interest. A binary descriptor may contain one or more binary digit, each digit being 1 or 0. The fragments were protonated according to the anticipated protonation states under physiological conditions; physico-chemical properties (2D descriptors) were calculated using MOE software. Provided that the property is quantitative, a binary descriptor comprising a string of binary integers is used to represent a pertinent numeric ranges of a given property; e.g. molecular weight can be described by ten binary integers, for instance below 50, 50 to 100, etc. The scoring function is Matthews' Correlation Coefficient (MCC).The second method used is the Iterative Stochastic Elimination algorithm (ISE) that was adapted for molecular bioactivity indexing of chemicals, by transforming the problem to a combinatorial one with variables, values and interactions between different choices. It is based on optimization of ranges for a set of descriptors and on an optimal choice of sets of descriptors.

For further details on the application of ISE to obtain the best ranges from a set of descriptors and for the optimization process, see our previously reported studies [46], [49], [50]. In addition to constructing a “best filter” for evaluating each single molecule's potential to be a bioactive or not, ISE also forms a large set of “efficient filters” that are alternatives to the optimal solution, each of them being somewhat less successful than the optimal. The efficiency of discrimination is increased in general by employing a “combined filters approach”, which results in constructing the MBI. It is based on the assumption that a “best bioactive molecule” (i.e., one having more of the desired bioactive qualities) would pass more of the “filters”, while a “non-bioactive” would be one which passes a minimal number of filters. This assumption is the basis for constructing the MBI, which is composed of the contribution of the number of filters passed by a molecule to that molecule's overall “bioactivity” quality.
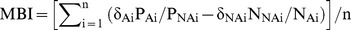
(I)


**Figure 2:**
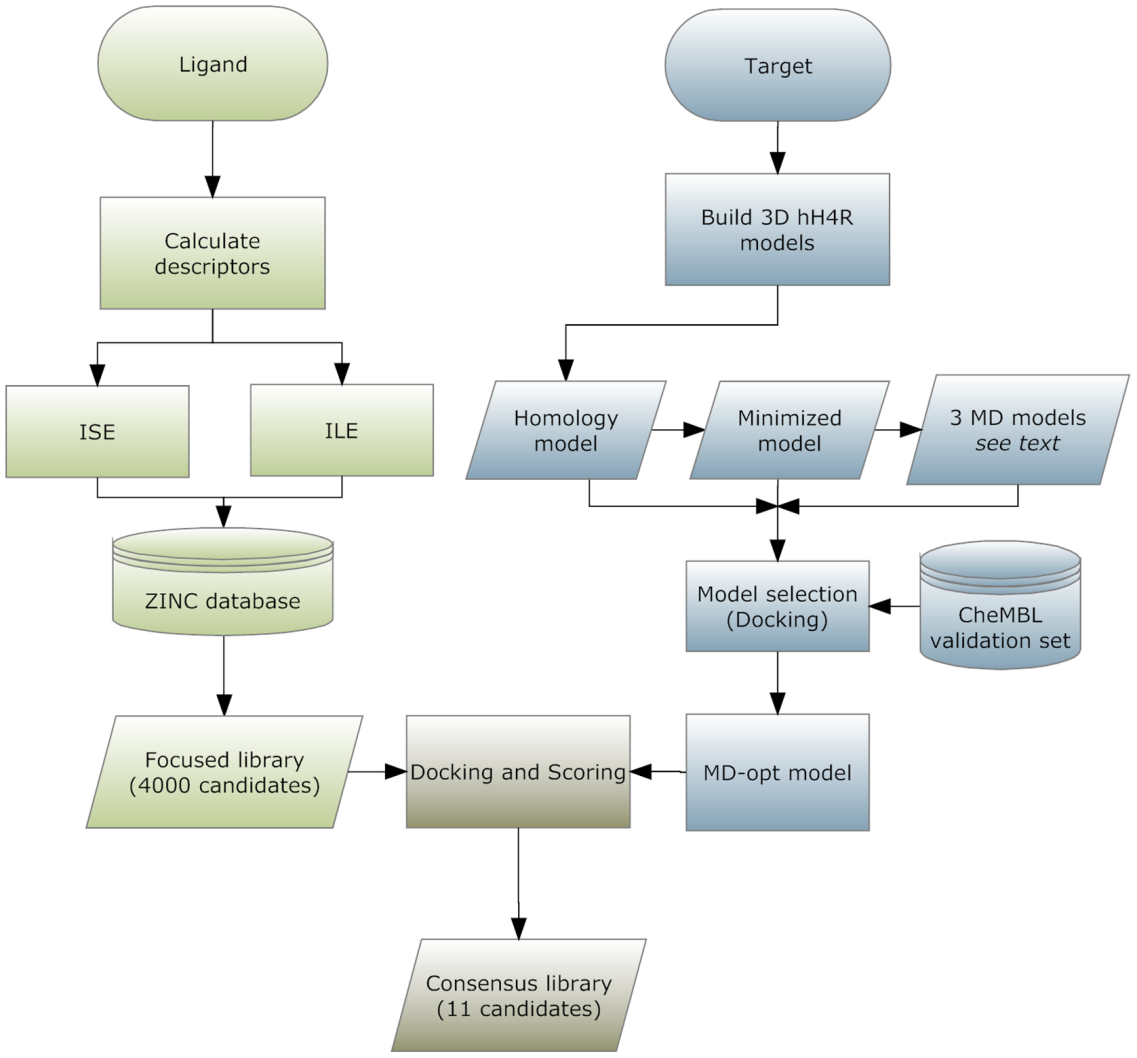
Flowchart of the combined ligand- and structure-based based approaches to index hH4R bioactive ligands.

In equation 1, the molecular bioactivity index (MBI) for a molecule is determined on the basis of a set of n filters and is constructed from all 4 numbers in two pairs – positives (%A) and false positives (which is 100 - %NA), as well as false negatives (100 - %A) and true negatives (%NA). The number of filters (n) could range from few to hundreds. The value of the delta function *δ_Ai_* is zero if the molecule is not active according to the currently calculated filter i, and one if it is active according to the filter. Similarly, the value of the delta function *δ_NAi_* is one if it is not active according to the currently calculated filter, and zero if it is an active according to the filter. P_Ai_ is the percentage of active molecules that are predicted to be “bioactives” according to filter i (“True positives”), while P_NAi_ is the percentage of false positives, *i.e.*, non-actives that are predicted to be bioactives according to filter i. N_Ai_ is the percent of actives identified to be non-bioactives according to the current filter (“False negatives”), and N_NAi_ is the percent of non-actives identified as such by the current filter, i.e., “True negatives”. The P_Ai_/P_NAi_ ratio, may be regarded as an “efficiency factor” of filter i for the bioactives, while the quotient N_Ai_/N_NAi_. is an “efficiency factor” for misidentifying non-bioactives. In Figure 2 a flowchart sketching the combined ligand-based and structure-based approach adopted is shown.

### hH_4_R 3D model generation

**Figure 3:**
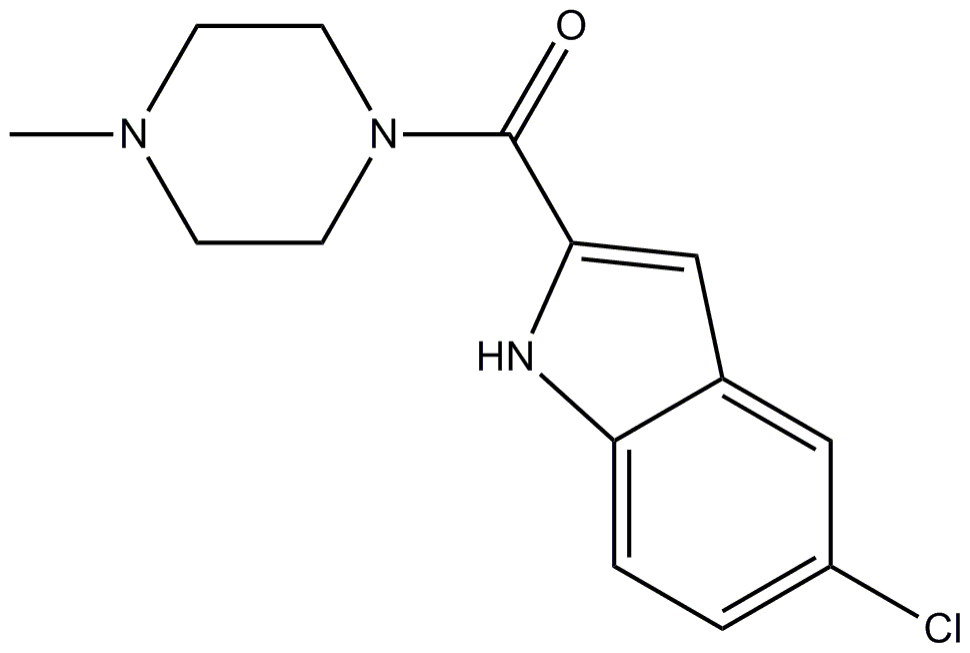
Structure of JNJ-777120, selective ligand of hH_4_R.

The crystal structure of H1R (3RZE) was used as a template to construct hH_4_R [60]. After removal of all atoms extraneous to the receptor, dissimilar side chains were added by the SCWRL tool [61]. The sequence of hH4R was retrieved from GenBank (Q9H3N8) (http://www.uniprot.org/uniprot/). All missing residues were added manually. Side chains of Aspartic acid, Glutamic acid, Arginine, Lysine and Histidine were considered to be charged (non-opt model). The obtained model was then minimized to relax the structure and remove steric bumps. The minimizations were performed by 2000 steps of steepest descent followed by 8000 step of conjugate gradient. Subsequently a short equilibration (100 ps) has been performed into water environment, adopting the same parameters adopted in the production runs. During equilibration, the intracellular loop 3 (Y198-E294) folded from a random coil to an α-helix conformation. To further improve the quality of the hH4R structure, a third model, based on the previously minimized structure, was assembled by inserting the min-opt model into a 1,2-dioleoyl-sn-glycero-3-phosphatidylcholine (DOPC) lipid bilayer. The membrane-embedded receptor was then equilibrated by running a series of fully atomistic MD steps. Unfortunately, during the MD simulations, the structure of the hH_4_R receptor collapsed, likely due to the lateral pressure applied by the lipid bilayer. Therefore, we filled the binding site with a known selective ligand (JNJ7777120), (see figure 3) which was presumed to counterbalance the effects of the lipid bilayer. The JNJ7777120 compound(chemical structure shown below) was built and optimized using the software SYBYL-X 2.0 (http://www.tripos.com). The JNJ7777120 parameters were determined by ANTECHAMBER 1.27 [62] using the General Amber Force Field (GAFF) [19]. Partial atomic charges were calculated using theAM1- BCC method [63], [64]. During all simulation of the second model we restrained distances between JNJ7777120 and hH4 amino acid E^5.46^ and D^3.32^ with a harmonic distance restrain. Finally, in the third model, the compound JNJ7777120 was free to move into the hH_4_R cavity.

All Molecular Dynamics simulations were carried out using NAMD7 [65] software and the CHARMM27 force field [66]. All hH_4_R models were embedded in DOPC lipid bilayer and solvated with water. Next, NaCl counter ions were added up to an estimated ionic strength of 0.15 mM. The final box was large enough to contain all of the protein atoms at the end of simulation. The size of the receptor/membrane/water system was 125×125×150 Å with approximately 187000 atoms. TIP3P was used as an explicit water model; the dielectric constant (ε) is 1, and non covalent interactions up to 1–4 are considered. Cut-off values for non-bonded interactions (Coulomb and Van der Waals forces) and for the switching function are 10 and 9 Å respectively. Langeving dynamics were used throughout all simulations. The SHAKE algorithm was used to fix the length of covalent hydrogen bonds. In all simulations we adopted standard periodic boundary conditions (PBC), and computed long-range interactions by the Particle Edwald Mesh method. The time-step was fixed at 2 ps, and the ‘rigid bond’ NAMD algorithm was used.

In the membrane system lipid tails were firstly energy-minimised (protein, water, counter ions and phospholipidic head were frozen during this minimization step) and gradually heated up to 300 K. After lipid tails optimization, we fixed the whole system with the exception of the receptor. Then the membrane-embedded enzyme was energy-minimised (conjugate gradient) and gradually heated up to 300 K and equilibrated for 400 ps. After the minimization step productive MD runs were launched. The following models of the hH_4_R were validated by docking studies: i) the non-optimized 3D structure of the receptor derived from homology modeling, ii) the same 3D structure optimized by minimization steps and iii) a series of 3D structures optimized by MD simulations. In this last case, the model with the ligand JNJ7777120 inserted and restrained into the pocket (named MD-opt model) provided the best results and was chosen for comparison with the homology (non-opt model) and the energy-minimized model (min-opt model). These models were tested for their ability to separate active ligands (i.e. hH_4_R antagonists) from a set of randomly selected inactive compounds. The docking results were analyzed using a dedicated scoring function by an automatic data processing script. The cavity volumes were obtained with CAVER software [67]. Hence, a spherical probe with a 1.4 Å diameter rolled inside the receptor, searching and highlighting all the cavities into the active site of the protein.

### Molecular Docking

The docking program Autodock [68] version 4.2 was managed by a Perl automation script. The binding pocket was extracted from the structure of the whole 7TM domain by taking a 19 Å radius around the center of the histamine binding site, in order to maintain reasonable CPU running time while keeping sufficient docking accuracy. The docking grid was set to include the extracted receptor domain, including the extra cellular loops and was approximately (37×35×41) Å on the X, Y and Z axes respectively. The genetic algorithm output size was set to 16 docked poses. The set of 78 active ligands indicated in the method section was filtered for diversity using the Tanimoto similarity measure [69] (S<0.7), which resulted in 46 diverse ligands denoted the “active set”. The set of random compounds was extracted from the ZINC database of commercially available compounds (http://zinc.docking.org/). The random compounds were filtered to ensure diversity (S<0.2) and to obey Lipinsky rule of five. Two random sets were used: one with 50 compounds, to match the size of the active set and another one with 137 compounds. An independent validation set was further retrieved from the chEMBL [70] database based on published KI and IC_50_ values. The set was filtered by diversity, both intra-wise (within set similarity) and inter-wise (compared to the set of active compounds already used). Filtering resulted in a set of 56 active compounds. In parallel, a focused library of putative hH_4_R binders was computed *in-silico* using a descriptor based approach (as described in the chemoinformatic section above). The total size of the focused library was 872 compounds rated highly by both ILE and ISE based models. All compounds were protonated using a pH values of 2.5 to maintain positively charged groups on Nitrogen atoms.

The docked poses were analyzed by two consecutive filters:

- A filter based on the calculated electrostatic energy of the docked pose. The motivation for using the electrostatic energy relies on the fact that two binding site residues, D^3.32^ (TM3) and E^5.46^ (TM5), which are critically involved in histamine binding, are both negatively charged in physiological pH. Therefore, any interaction in the binding site must have a strong electrostatic interaction between the ligand and at least one of these residues. The electrostatic energies were extracted automatically from the Autodock docking log files using a script written in Perl. The exact thresholds of the filter were defined separately for each inspected receptor model, based on plotting and visual inspection of docking results. Additionally, k-means clustering algorithm [71] was applied to automatically find clusters based on electrostatic energy and was found to be in a good agreement with the visual inspection of the plots.
- A conformation positional filter which relies on the calculated distance between the heavy polar atoms of the ligand and the carboxylic acid side chain group of the binding residues D^3.32^ and E^5.46^. The distance threshold was set to 4 Å. Any atom-atom distance below the threshold was considered a putative interaction between the ligand and the receptor binding residue. Three conformational filter scores were defined: zero (no interaction), one (possible interaction with just one of the receptor reference residues) and two (possible interaction with both receptor reference residues).

## Results and Discussion

### Ligand-based chemoinformatics models

**Figure 4:**
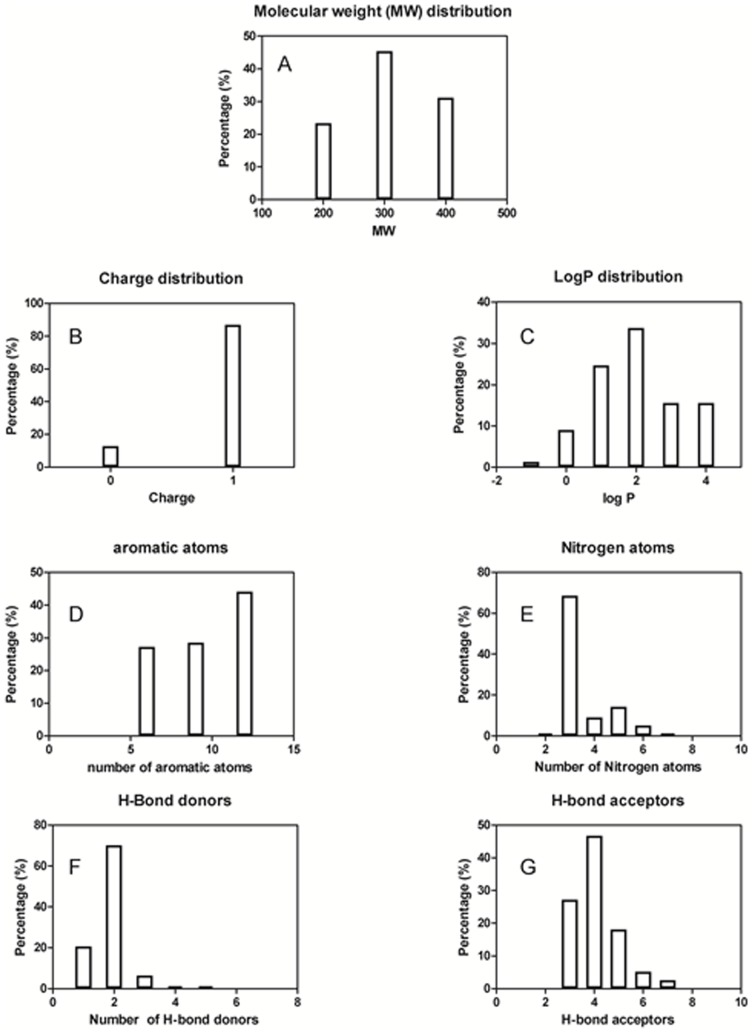
Distribution plots of several physico-chemical properties of the set of 78 hH_4_R antagonists: Molecular weight distribution (A), Total charge (B), Log P values (C), Number of aromatic atoms (D), number of nitrogen atoms (E), Number of H-bond donors [lip_don] (F), Number of H-bond acceptors [lip_acc] (G).

Figure 4 shows the distribution plots of several physico-chemical properties of the set of 78 hH_4_R antagonists. Figure 4A shows that the mean molecular weight is just above 200 Da. All the antagonists contain more than one nitrogen (Figure 4E) and have functional groups that could be able to form strong specific interactions like hydrogen bonds and electrostatic interactions [72], [73], [74] (Figure 4F &4G).

**Figure 5:**
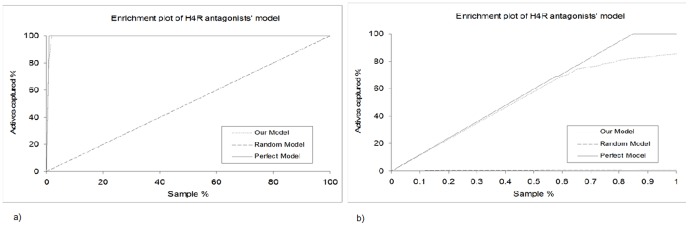
Enrichment plot (a) and Enrichment plot of the highest indexed 1% chemicals (b).

Interesting complementarities can be found between the properties of hH_4_R antagonists and their receptor binding pocket. The antagonists contain at least one positively charged groups, preferably two (Figure 4B). In the hH_4_R binding pocket, these features are complementary to two negatively charged residues, namely Asp94 in helix III (D^3.32^) and Glu182 in helix V (E^5.46^), known to be important for the interaction of high affinity ligands. As well, many hH_4_R antagonists can bind the receptor with a high ligand-lipophilic efficiency (this affinity is diminished with logP). This also explains the logP distribution of hH_4_R antagonists (Figure 4C). We have found that hH_4_R antagonists are drug-like, 100% obey Lipinski rule of 5. Drug-like, according to the Lipinski rule of 5, states that orally bio available molecules are more likely to have H-bond donors ≤5, H-bond acceptors ≤10, molecular weight ≤500 and log P≤5. As well, all hH_4_R antagonists collected from literature were found to obey the rules of Oprea for lead-likeness. Oprea rules for lead-likeness states that lead molecules are more likely to have molecular weight ≤450, log P ranges between −3.5 and 4.5, rings ≤4, non-terminal single bonds ≤10, hydrogen bond donors ≤5 and hydrogen bond acceptors ≤8. They were derived by an analysis of 96 drugs and leads from which they were derived. The ISE based model generated forty eight unique filters. The MCC of the filters range between 0.6 and 0.87. One of the filters is 3-rules based model which states thathH_4_R antagonists compared to ZINC drug-like chemicals are more likely to have: total charge equal +1; 3 ≤N atoms ≤7; fractional negative VDW surface area ≤0.42. More than 87% and less than 5% of the hH_4_R antagonists and ZINC drug-like chemicals respectively obey the three rules-based model described before. The MCC of this filter alone is 0.82. The combined ligand-based approaches (ISE & ILE) produced highly efficient model for indexing chemicals for their hH_4_R antagonism (see figure 5). More than 80% of the actives were captured at the top scored screened set (figure 5b).

#### Generating a reliable 3D models of hH4 for drug discovery

**Figure 6:**
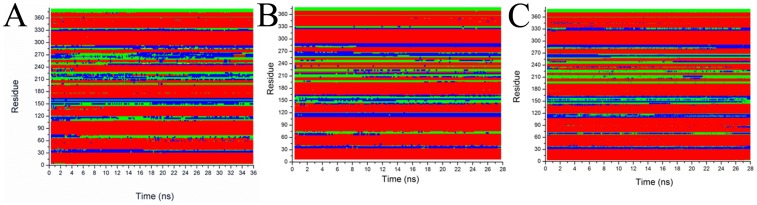
Time evolution of hH_4_R secondary structure content during equilibration in the absence (panel A) and in the presence (panel B) of the ligand JNJ7777120. Panel C reports the time evolution of the secondary structure of hH_4_R in the presence of the ligand restrained as described in the methods section. Color codes are: red/helix, green/coils and blue/turns.

The set-up of an equilibrated hH_4_R structure in a membrane environment is a necessary prerequisite for subsequent docking calculations. During the equilibration MD runs (36 ns) the structural integrity of the receptor was monitored by analyzing the evolution of its secondary structure versus time (see Figure 6 panel A). The seven helices of the trans-membrane domain are almost unaltered over the whole simulation. On the contrary, residues spanning from 200 to 290, belonging to flexible domain of the receptor, exhibit a highly dynamic equilibrium between helical, random coil and turn structural motifs. Residues 35, 75, 110, 150 and 330 remain unstructured.

**Figure 7:**
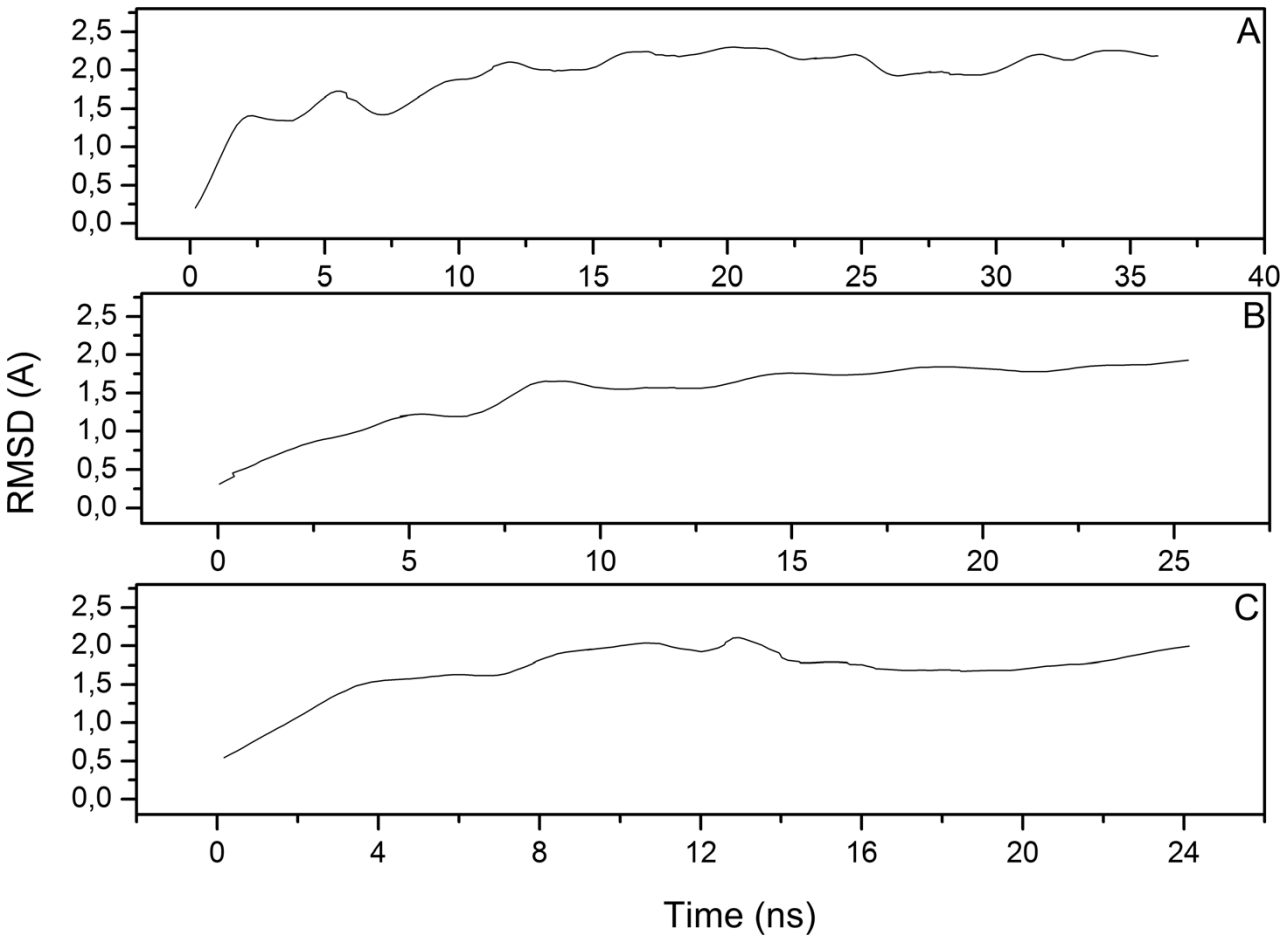
RMSD for backbone atoms of hH4R in membrane environment (panel A); with restrained ligand (panel B) and with unrestrained ligand (panel C). RMSD has been calculated considering starting frame as reference.

As mentioned before, MD runs on the minimized hH_4_R model resulted in a different size of the binding cavity, as reported in table 1 and 2 likely due to the lateral pressure applied by the lipid bilayer. To prevent the crumple of the binding pocket we inserted into the binding pocket a known ligand of the hH_4_R receptor (JNJ7777120). The presence of the ligand does not alter the secondary structure content of hH_4_R as reported in figure 6 panel B, and C. Only small differences may be observed when the position of the ligand is restrained; in particular residues near D^3.32^ lose partially their helicity. The RMSD of hH_4_R without ligand (Figure 7, panel A) reaches a value of 2 Å at about 10 ns and then fluctuates at 11 Å with very small oscillations indicating that the structure is equilibrated.

**Table 1:** Amino acids involved in the binding pocket of the four models here studied.

Homology model	Equilibrated model	with UnRestrained ligand	with Restrained ligand
94 ASP	91 LEU	90 TRP	94 ASP
95 TYR	94 ASP	94 ASP	95 TYR
98 CYS	95 TYR	95 TYR	96 LEU
99 THR	96 LEU	96 LEU	98 CYS
147 ASN	98 CYS	98 CYS	99 THR
168 PHE	99 THR	99 THR	147 ASN
169 PHE	146 VAL	100 ALA	150 MET
182 GLU	147 ASN	103 TYR	158 LYS
316 TRP	150 MET	143 ALA	160 GLU
319 TYR	166 PRO	146 VAL	161 GLY
	168 PHE	147 ASN	162 SER
	174 ILE	168 PHE	163 GLU
	175 LEU	175 LEU	164 CYS
	178 THR	178 THR	165 GLU
	179 SER	179 SER	166 PRO
	182 GLU	182 GLU	168 PHE
	183 PHE	183 PHE	169 PHE
	319 TYR	319 TYR	174 ILE
	320 SER	320 SER	175 LEU
	321 LEU	323 THR	178 THR
	323 THR	344 PHE	179 SER
	324 ILE	347 GLN	182 GLU
	327 SER		183 PHE
	347 GLN		319 TYR
			320 SER
			321 LEU
			322 PHE
			323 THR
			324 ILE
			326 LEU
			327 SER
			330 SER
			331 SER
			333 THR
			334 GLY
			335 PRO
			340 TYR

We evidenced in bold, amino acids common to all four active sites.

**Table 2:** Conformational binding pocket analysis of the four constructed models.

	Homology model (model before minimization & MD)	MD Model 1 of hH4R with restrained ligand	MD Model of hH4R with unrestrained ligand	MD Model of hH4R without ligand
Cavity volume (A^3^)	304	658	500	550

A similar behavior is also observed in the presence of the restrained ligand, with constant RMSD values of 1.8 Å observed at about 10 ns. Conversely, the RMSD values of the hH_4_R model simulated in the presence of unrestrained ligand reaches a constant value of about 1.7 Å after 8 ns. These results ensure that all structures are fully equilibrated by MD runs.

**Figure 8:**
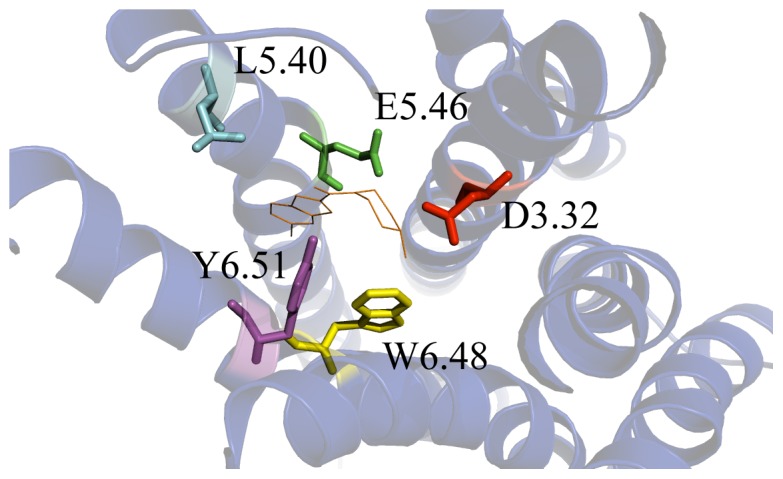
Cartoon representations of the hH4R models with unrestrained ligand JNJ7777120 at the center of the figure (orange lines). Residues involved in H4 binding, with JNJ7777120 are evidenced as sticks.

In order to produce a suitable 3D structure for docking calculations we averaged all the MD frames over last 100 ps, and minimized the obtained structures. As previously reported, the residues D^3.32^ and E^5.46^ are critically involved in histamine binding [75], [76]. Therefore their conformations may indicate if the overall protein structures could be used for reliable docking calculations. In figure 8, the MD-optimized structures of hH_4_R indicating both amino acids are represented. It is shown that both amino acids point toward the inner core of the receptor, indicating that all the simulated structures, albeit with subtle conformational changes, adopt conformations that are suitable for docking studies. Moreover the pose of JNJ7777120 ligand into the active site of our model is in accordance with previous reported results [72], [77], [78]. Our findings show that at least one atom of the ligand JNJ7777120 is located at distance ≤3 Å respect to D^3.32^ (Asp94), E^5.46^ (Glu182), L^5.40^ (Leu175), Y^6.51^ (Tyr319) and W^6.48^ (Trp316), as reported in figure 8.

**Figure 9:**
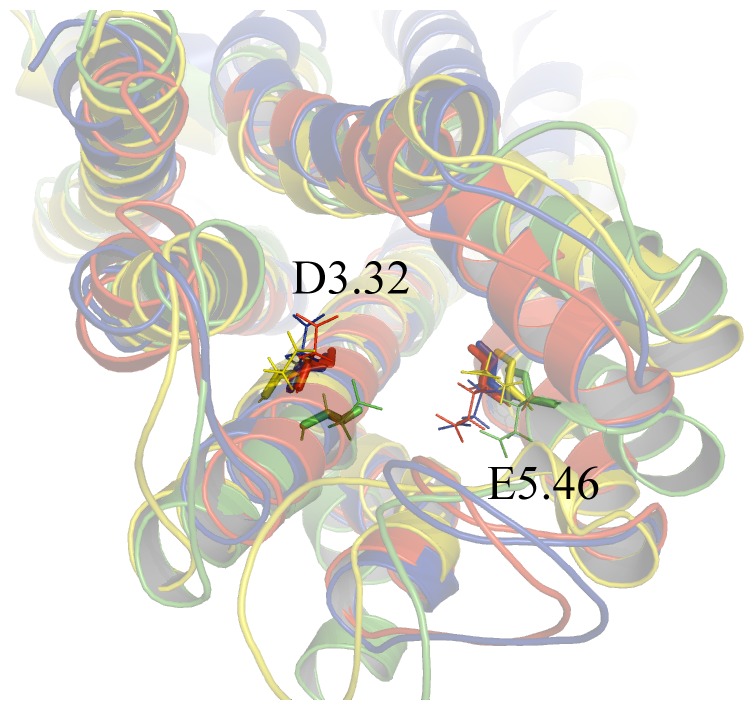
Cartoon representations of the four hH4R models here studied: i) homology model (yellow), equilibrated model (blue), with restrained ligand (green), with unrestrained ligand (red). Residues Asp94 (D3.32) and Glu82 (E5.46) are evidenced as sticks.

A comparison of the four models adopted here is also reported in figure 9. The position of the D^3.32^ residue is similar in the equilibrated and in the unrestrained ligand model. Conversely, in the ligand restrained model it appears to be shifted, likely due to the above mentioned helicity loosening. The conformation of D^3.32^ in the homology model, albeit close to the red and blue structures, points to a rather different direction.

The conformation of the E^5.46^ residue is similar in all the four structures, but, in the ligand restrained model, E^5.46^ is translated respect to the other models. Moreover, the E^5.46^ residue in the homology model, points to a different direction if compared to the other three structures. These diversities in the active site geometry reflect also a different cavity size. Furthermore, all residues that form the binding site in the homology model are included, also, in the active pocket of the three other models studied, with the exception of the TRP 316 and PHE 169 (see Table 1). Altogether, these data suggests that a different choice of the structural model may imply significant differences in the cavity volume of the active site and, in turn, in its supposed ability to accommodate antagonists. Table 2 reports the active site cavity volumes of the four models examined in this study: it is evidenced that the structure equilibrated after MD simulations performed in the presence of a restrained ligand shows the largest cavity size (658 Å^3^), and therefore, it is conceivable that it could represent the most reliable model to be used for further docking studies.

### Docking & Scoring

After constructing the efficient ligands-based model, we need to select the best hH_4_R model to be used for docking purposes by a proper scoring procedure. Three different 3D models have been checked by docking studies:

- Homology based model without optimizations (aka “homology model”).
- Homology based model obtained after energy minimization only (aka “min-opt model”).
- Homology based model obtained after molecular dynamics (MD)and minimization (aka “MD-opt model”).

As already pointed out, a comparison of the three MD optimized models (i.e. equilibrated model in the absence of ligand, with restrained ligand and with unrestrained ligand) have shown that the MD model optimized with a restrained ligand (MD-opt model) exhibited the best binding pocket accessibility in docking simulations. Therefore it is used for further docking studies as a representative of the three MD optimized models (atomic coordinates are presented in File S3).

**Figure 10:**
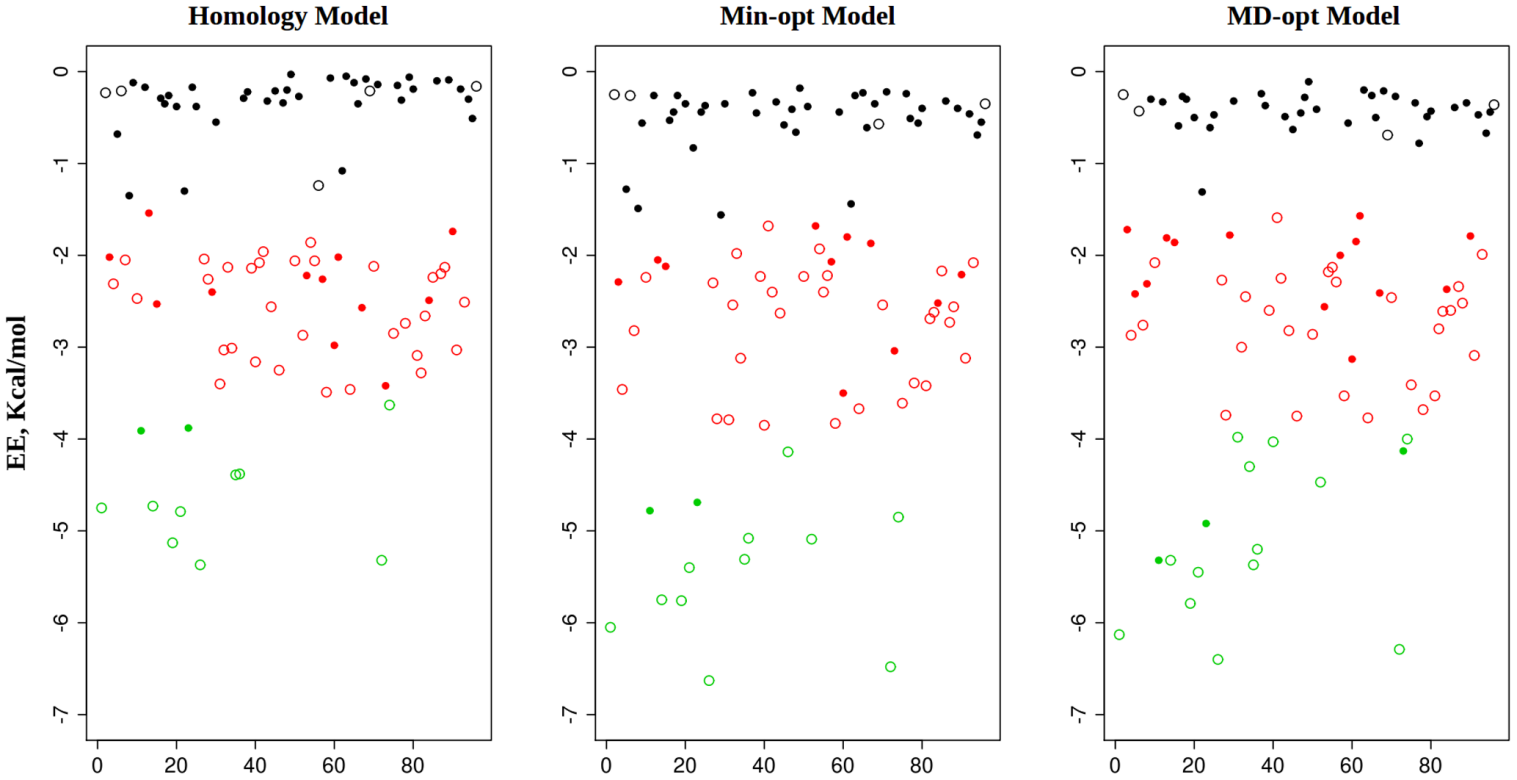
Computed electrostatic energy (EE) of the test set compounds docking simulations across three model versions (namely: the homology model, min-opt and MD-opt models). For the sake of clarity, only the best docked pose was kept for each compound, to maintain clarity. Open circles denote compounds with known binding activity (i.e. our positive control group). Closed circles denote random compounds (i.e. non-binders, negative control group). Colors denote group membership by K-means clustering. The plots show a distinct separation between two compound groups around −1.1 Kcal/mol and yet another one around −4 Kcal/mol. This pattern is observed across the model versions, with slightly lower EE values visible in the optimized models.

The docking test set (see methods) was docked into the extracted TM receptor domain of each model and the results of computed docked energies are presented in Figure 10, which shows a distinct gradient of energies and a clear separation into three groups, visible around −1.1 and −4 Kcal/mol, detected by unsupervised K-means clustering. These electrostatic energy values were next used as two separate filtering categories to compute the TP/FP enrichment factors and compare between the different models. Additional filtering by conformation positional closeness to the key binding site residues has resulted in a meaningful enrichment of active ligands compared to the non-active random set. Since the same compounds were docked into the receptor domain using exactly the same simulation conditions, the percentage of docked poses with closeness to both catalytic residues inside the binding pocket (i.e. distance score = 2) indicates that there is a difference in binding site accessibility between the different models: the best overall binding pocket accessibility is observed in the min-opt model, but in terms of TP/FP enrichment it is slightly inferior to the homology model but better than the MD-opt model. Adding the computed docked electrostatic energy as a filter and using just the lowest energy cluster as a filtering threshold improves considerably the enrichment factors in all models, but with price of increased false negative rates: 16 out of 46 (∼35%) active compounds passed our filters in the non-opt model, 13 (∼28%) in the min-opt model and just 8 (∼17%) in the MD-opt model. Considering both clusters (i.e. all poses in the lowest and second lowest electrostatic energy clusters) and a geometrical distance score = 2, generally leads to lower false negative rates. Since our preliminary tests have shown the two filters (calculated electrostatic energy and geometric distance scores), to be practically orthogonal, they can be combined in a useful way without a redundancy in the scoring model.

Docking results of the three different models are shown in table 3. Two electrostatic energy thresholds were set to values of −1.1 Kcal/mol and −4 Kcal/mol as previously found by the K-means clustering. The conformation distance score reflects geometric “closeness” and possible interaction with two negatively charged binding site residues. Two groups were compared: active ligands and a random set compound set (containing 46 and 50 compounds respectively). The enrichment factor is the ratio between the active and random compounds left following each filtering step. Two sets of chemicals were used to validate the hH_4_R model by external test sets: one including a new set of ligands with known activity, extracted from the chEMBL database and containing 56 ligands (aka “chembl verification set”) and the set of candidates derived from the chemoinformatics indexing which contains 872 diverse targeted binders (aka “focused set”). Both sets were compared to a set of 137 random compounds extracted from ZINC and regarded as a negative control set in this procedure. All sets were docked using the same protocol and processed the same way. Their performance results are described below.

**Table 3:** The docking results of the three models are shown here.

	Homology model	Min-opt model	MD-opt model
Distance score = 1	67%	84%	91%
Distance score = 2	38%	44%	16%
Enrichment (lowest EE cluster+distance score = 2)	16 (85%)	13 (87%)	4 (89%)
Enrichment (two EE clusters+distance score = 2)	3.3 (33%)	2.5 (16%)	2.3 (18%)

Two electrostatic energy thresholds were set to values of −1.1 Kcal/mol and −4 Kcal/mol as previously found by the K-means clustering. The conformation distance score reflects geometric closeness and possible interactions with the negatively charged binding site residues (D^3.32^and E^5.46^. Two groups were compared: active ligands and a random, set (containing 46 actives and 50 non-active compounds respectively). The enrichment factor is the ratio between the active and random compounds left following each filtering step. Percent in brackets denote the false negative rate for each model and configuration.

Homology model = H4 receptor model before minimization & MD.

Min-opt model = H4 receptor model after minimization only.

MD-opt model = H4 receptor model after minimization and MD.

Distance score = 1 means closeness to one of the following residues Asp-92 and Glu-182, while distance score = 2 means closeness to both residues.

### chEMBL verification set

An initial inspection of the docking results has shown that very few docked poses (about 1% of the poses, compared with roughly 16% of poses in the previous test set evaluation) had low enough calculated electrostatic energy to be classed in the lowest energy cluster, defined previously using the test set as lower than −3.16 Kcal/mol. Therefore the alternative scoring strategy of using the two lowest electrostatic energy clusters and the geometrical closeness score was used. The random sample set of non-binders was randomly sampled in a 20-fold sub-sampling procedure and each sub set was compared in turn to the set of active ligands. The mean amount of compounds passing the filters over all sub-sets was then used for calculating the enrichment factors. The results of filtering to retain active ligands are presented in Table 4.

**Table 4:** Docking results and enrichment factors for the chEMBL hH_4_R antagonists (external data set) and the focused library data sets.

Filtering Method:	Data set:	Verification Set		Focused Library	
		chEMBL Ligands	Random Set	Focused Set	Random Set
Two EE clusters+Distance score = {1,2}	Compounds Passed (%)	62.9	4.3	24.1	4.4
	Enrichment factor	14.6		5.5	
Two EE clusters+Distance score = 2	Compounds Passed (%)	14.8	0.9	7.7	0.7
	Enrichment factor	16.4		11	

Percent represents the fraction of total docked compounds which passed each filter. For enrichment calculations sub-sampling was used and the mean enrichment factors of the sampled groups are given.

### The focused library

**Figure 11:**
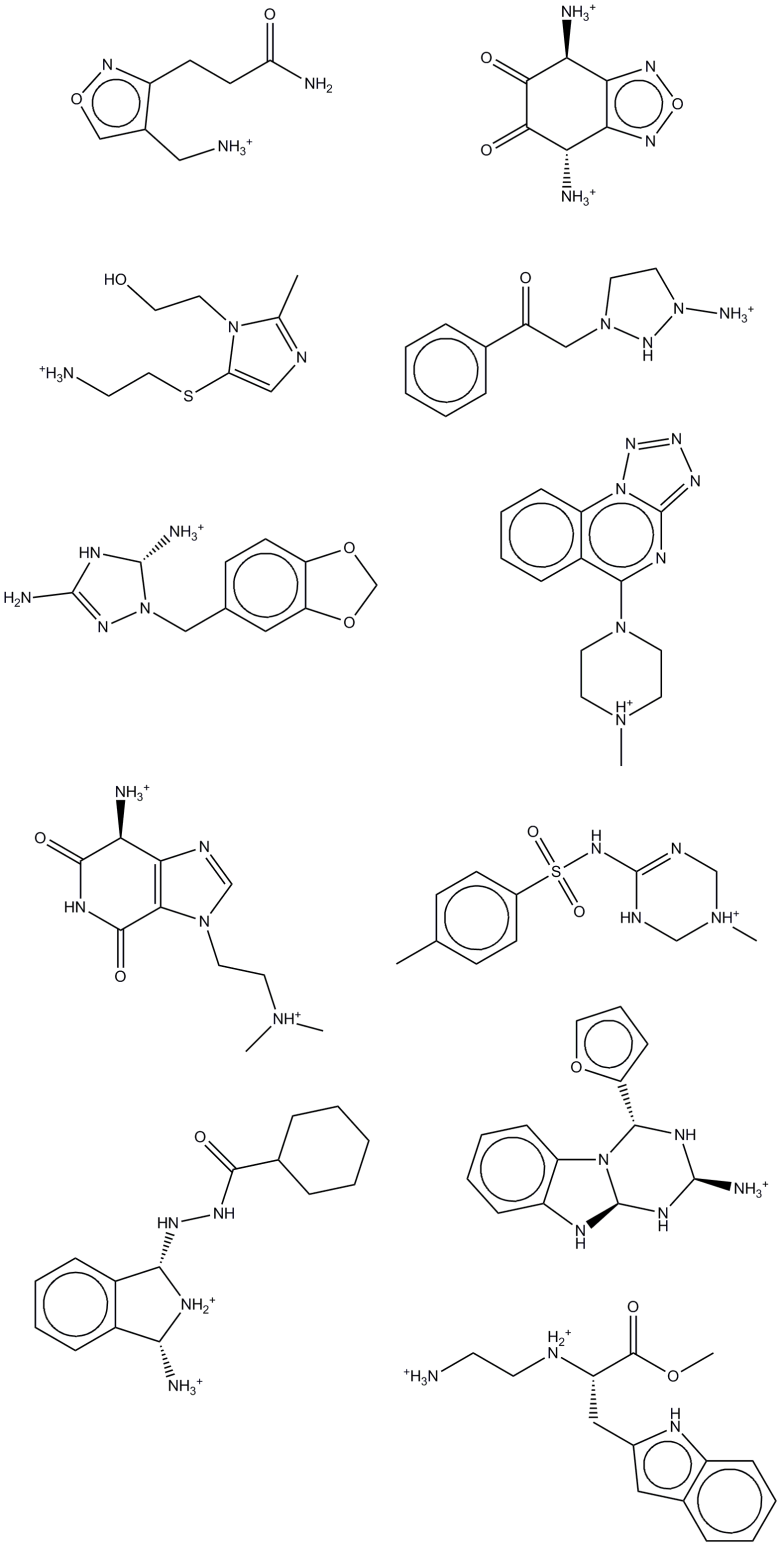
Consensus library of 11 candidates with top score.

**Figure 12:**
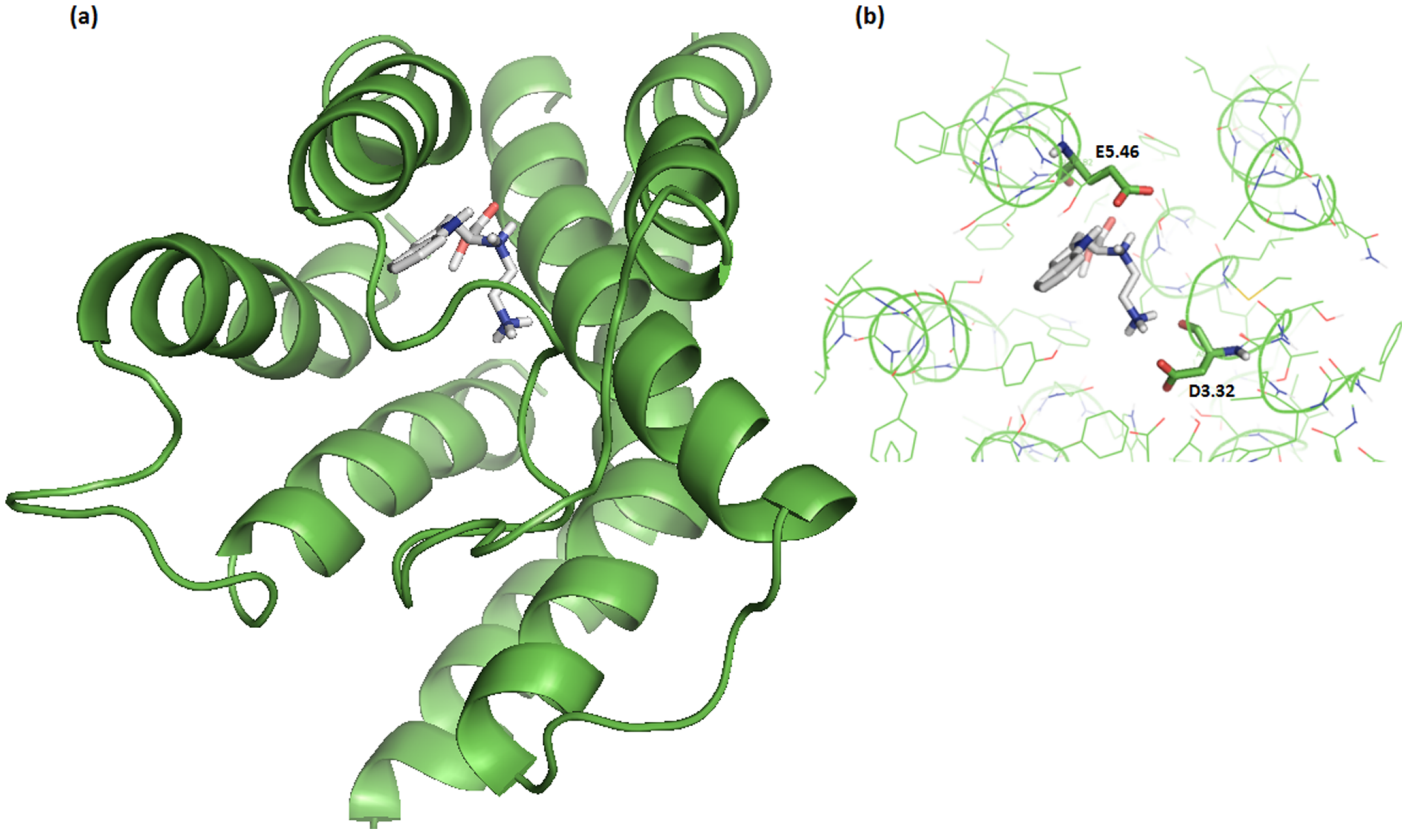
One of the binding poses of one of the top scored candidates. It interacts with both residues, D^3.32^ and E^5.46^ of the H_4_ receptor binding pocket, via salt bridge interactions.

An initial inspection of the docking results has shown that relatively few docked poses had low enough calculated electrostatic energy to be classed in the lowest energy cluster (about 4% of the docked poses).Therefore the alternative scoring strategy of using the two lowest energy clusters and a filtering based on geometrical closeness was used. A 100-fold random sub-sampling of the focused library compound space was done in order to have comparable amount of docked poses in each evaluation pass, so that exactly 137 docked compounds were compared at each iteration. The results of these iterated enrichment evaluations are presented in Table 4. The mean values of the sub-sampled groups were used to calculate an enrichment factor relative to the random compound set. Some of the best scoring hits are presented in Figure 11 (binding modes & energies of 11 hits are presented in File S4). The ligand binding pose of one of the top candidates is shown in figure 12.

Docking results and enrichment factors for the chEMBL hH_4_R antagonists and the focused library data sets are depicted in table 4. Percents represent the fraction of total docked compounds which passed each filter. For enrichment calculations sub-sampling was used and the mean enrichment factors of the sampled groups are given. Table 4 evidences that our combined ligand-based and structural approach is able to increase the hit rate in virtual high-throughput screening. In particular, ligand-based approaches allow screening large databases of chemicals in a highly fast and efficient way, reducing the number of potential candidates from millions of candidates to several thousands. The enrichment factor is 11 for the focused library compared to 16.4 of the external test set. About 7.7% of the chemicals in the focused library will pass both energy/positional filters and constitute the fraction of highly indexed potential candidates.

## Conclusions

The human histamine H_4_ receptor is an increasingly attractive drug target due to its relevance for the treatment of several inflammatory, allergic and autoimmune disorders, as well as for analgesic activity. There is still unmet need for discovery of hH_4_R antagonists and applying computerized techniques for virtual screening of large chemical databases could make the discovery process more efficient (shorten time and lower costs). In this paper a combined ligand-based and structure-based approach for indexing chemicals for their hH_4_R antagonism is reported. Firstly, two ligand-based chemoinformatics techniques, the Intelligent Learning Engine (ILE) and Iterative Stochastic Elimination approach (ISE), were utilized to screen the ZINC database and to pick ∼4000 chemicals highly indexed as H_4_R antagonists' candidates. Next, different hH4R structural homology models were made and their capability in differentiating between active and non-active H_4_R antagonists were examined by docking a validation set (extracted from the chEMBL database). For ranking the ligands and docked poses, a part of the AutoDock4 energy and particularly the electrostatic term, the filter of the ability to interact with D^3.32^ (TM3) and E^5.46^ (TM5) via hydrogen bonding/electrostatic interaction was taken into consideration. Among all the investigated models, a 3D hH4R structure modeled by extensive Molecular Dynamics simulation performed in a DOPC lipid membrane has been selected as the most efficient one. This last model was then chosen to screen the previously focused library obtained by applying the ligand-based approaches. A consensus library made of 11 drug candidates is finally reported and proposed as novel lead compounds. Our results suggest that a sequential combination of the ligands-based chemoinformatics techniques (ISE&ILE) with molecular modeling techniques has the potential to improve the success rate in discovering new biologically active compounds and increase the enrichment factors in a synergistic manner.

## Supporting Information

File S1 ‘S1_H4_active_literature_ligands_smi.xls’ including hH4R antagonists' dataset collected from literature.
(XLS)

File S2 ‘S2_hH4R_antagonists_diverse_from chEMBL.xls’ including hH4R binders extracted from chEMBL.
(XLS)

File S3 ‘S3_hH4R_model used for docking.pdb’ including the coordinates of the hH4R model used for docking purposes.
(PDB)

File S4 ‘S4_binding modes and energies.doc’ including binding modes and energies of the 11 bioactive candidates.
(DOC)
