# Supplementary material for: Sequential Application of Ligand and Structure Based Modeling Approaches to Index Chemicals for Their hH4R Antagonism
Source: PLoS One. 2014 Oct 16;9(10):e109340. doi: 10.1371/journal.pone.0109340 (PMC4199621; doi:10.1371/journal.pone.0109340)
Supplement: File S4 — ‘S4_binding modes and energies.doc’ including binding modes and energies of the 11 bioactive candidates. (DOC) [file pone.0109340.s004.doc]

SI Table 1. Calculated energy values for the test set of 46 active compounds and 11 candidate hits taken from the focused H4 receptor library. For each docked ligand, the pose with the best calculated electrostatic energy (EE) was taken.


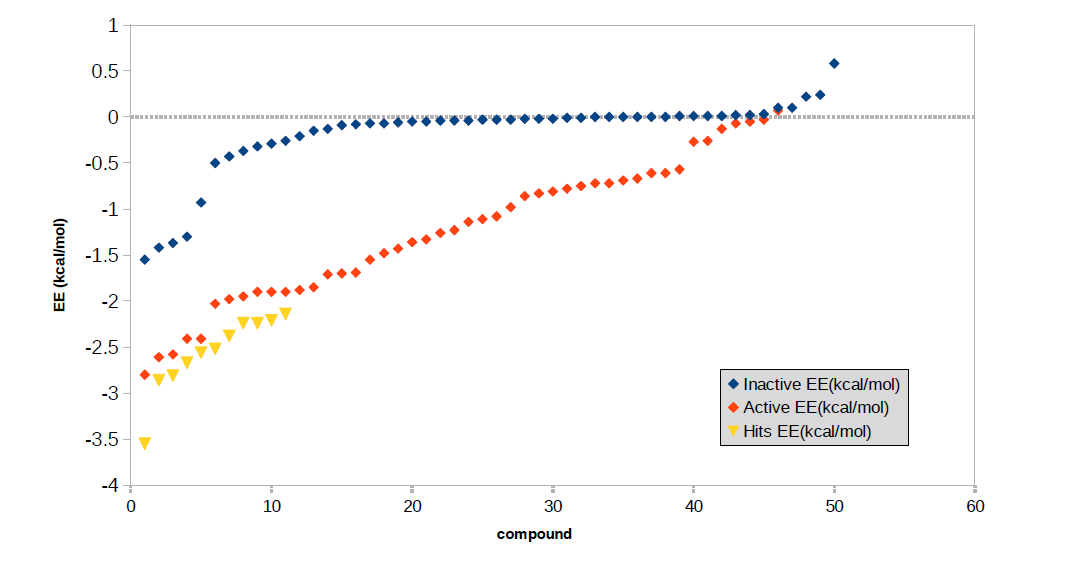


SI Figure 1. Calculated electrostatic energy (EE) for the 50 inactive test set (blue), 46 active test set (red) and 11 candidate hits (yellow) compounds. The candidate hits taken from the H4 receptor focused library show a distinct profile lower than the set of active compounds.


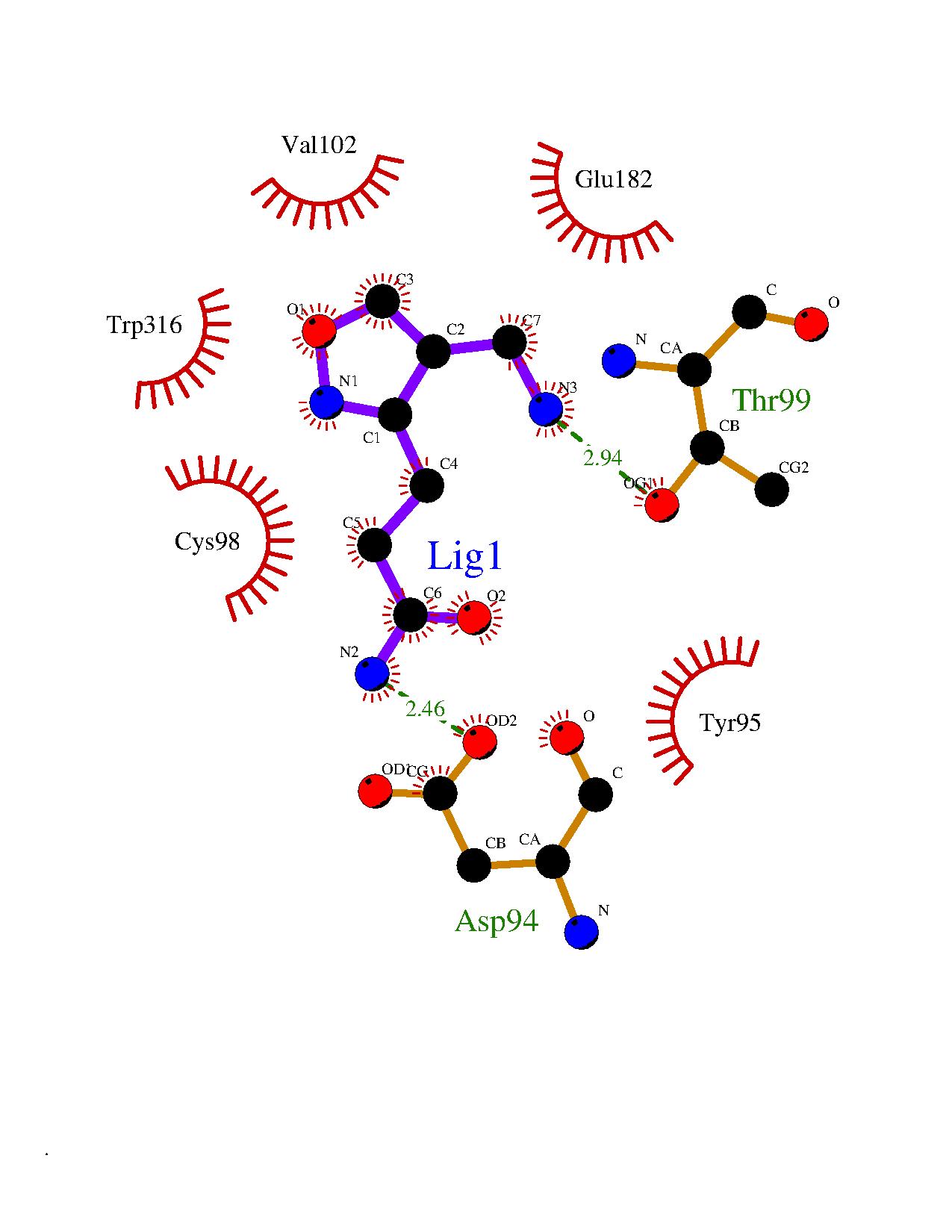


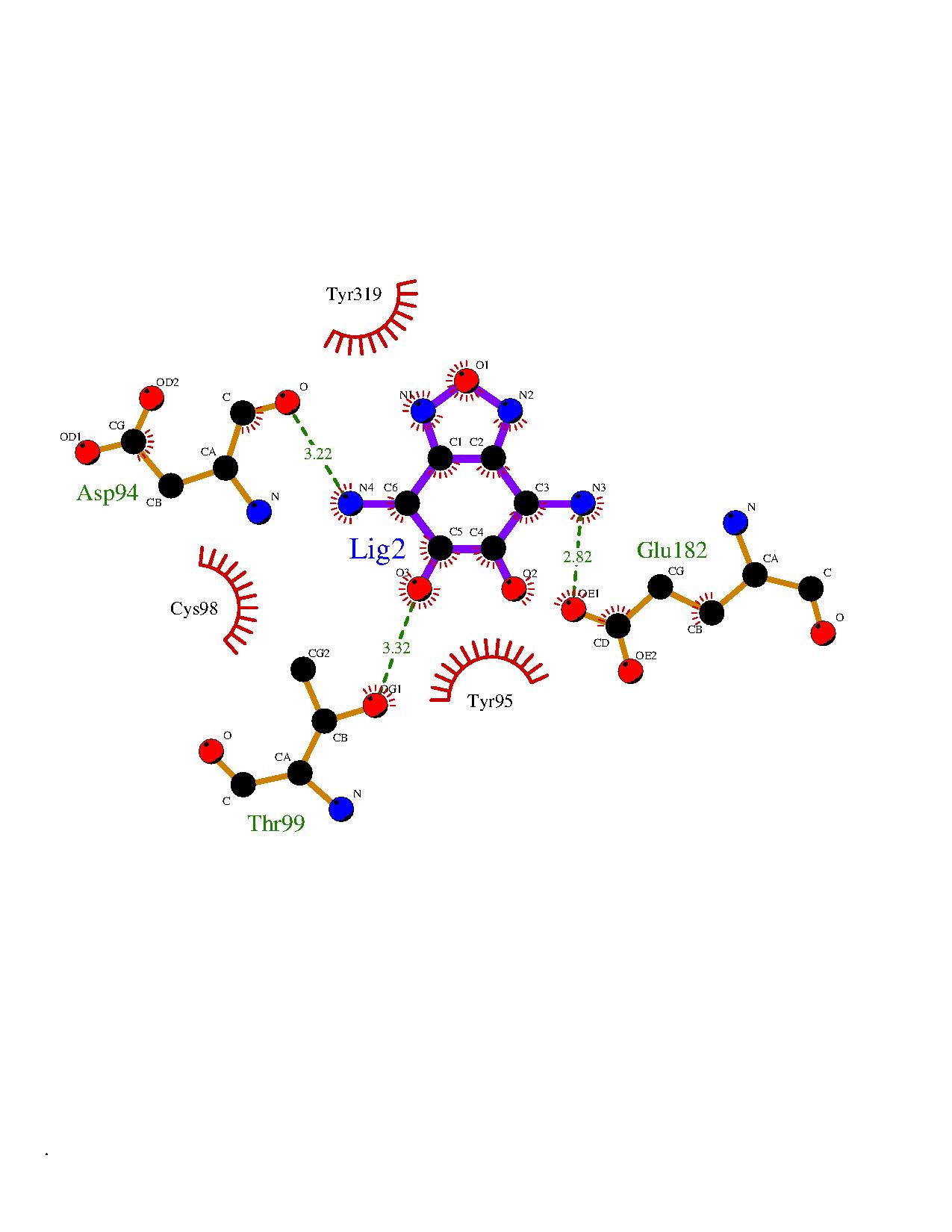


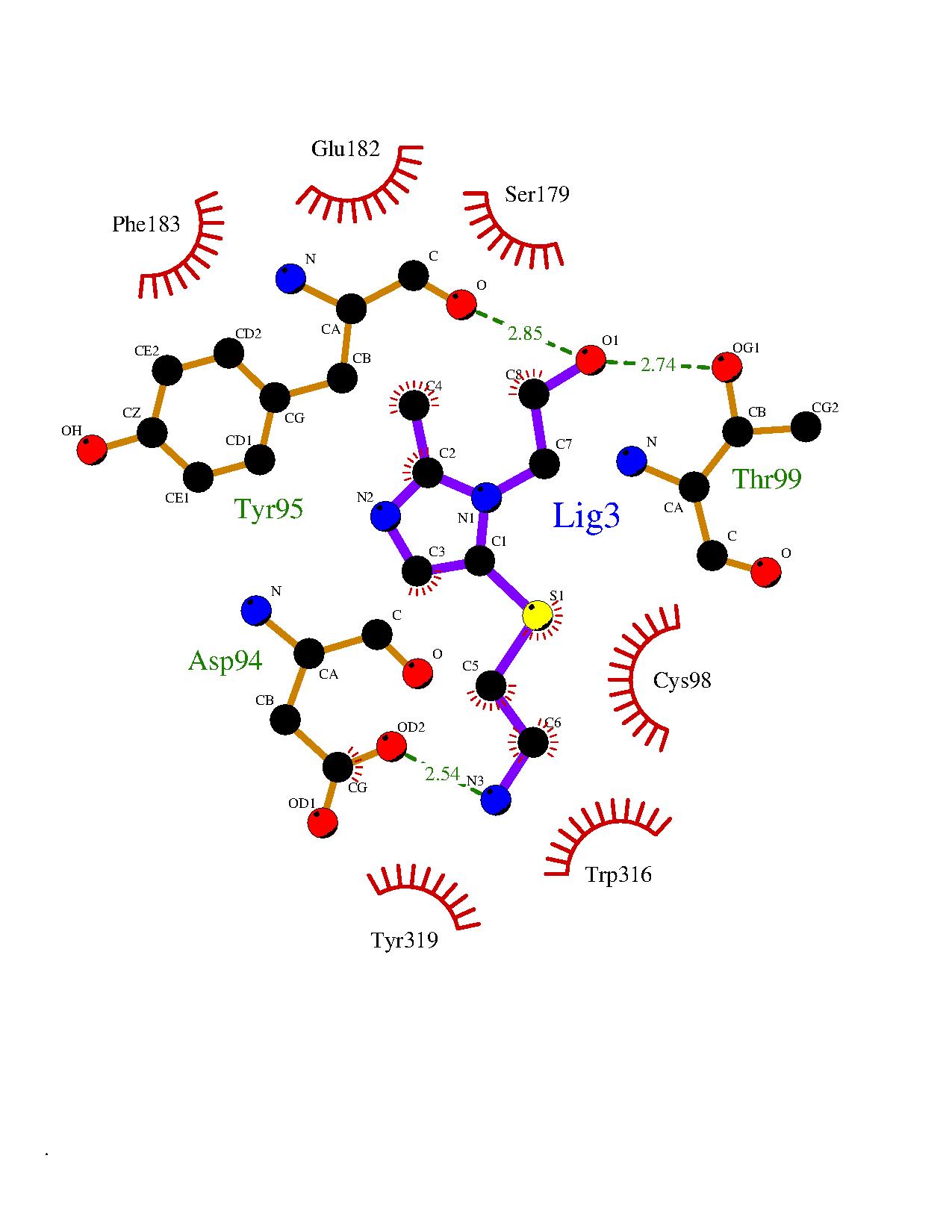


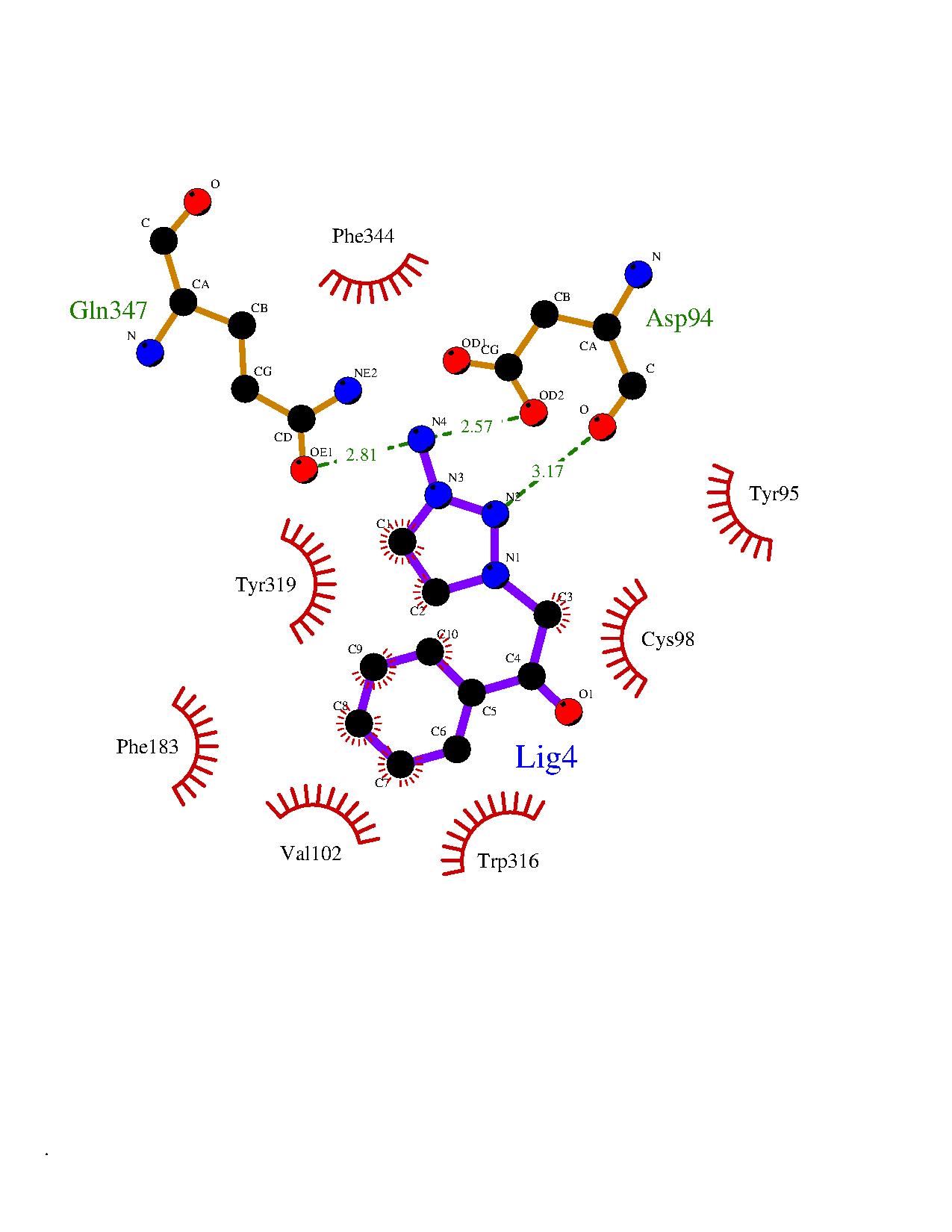


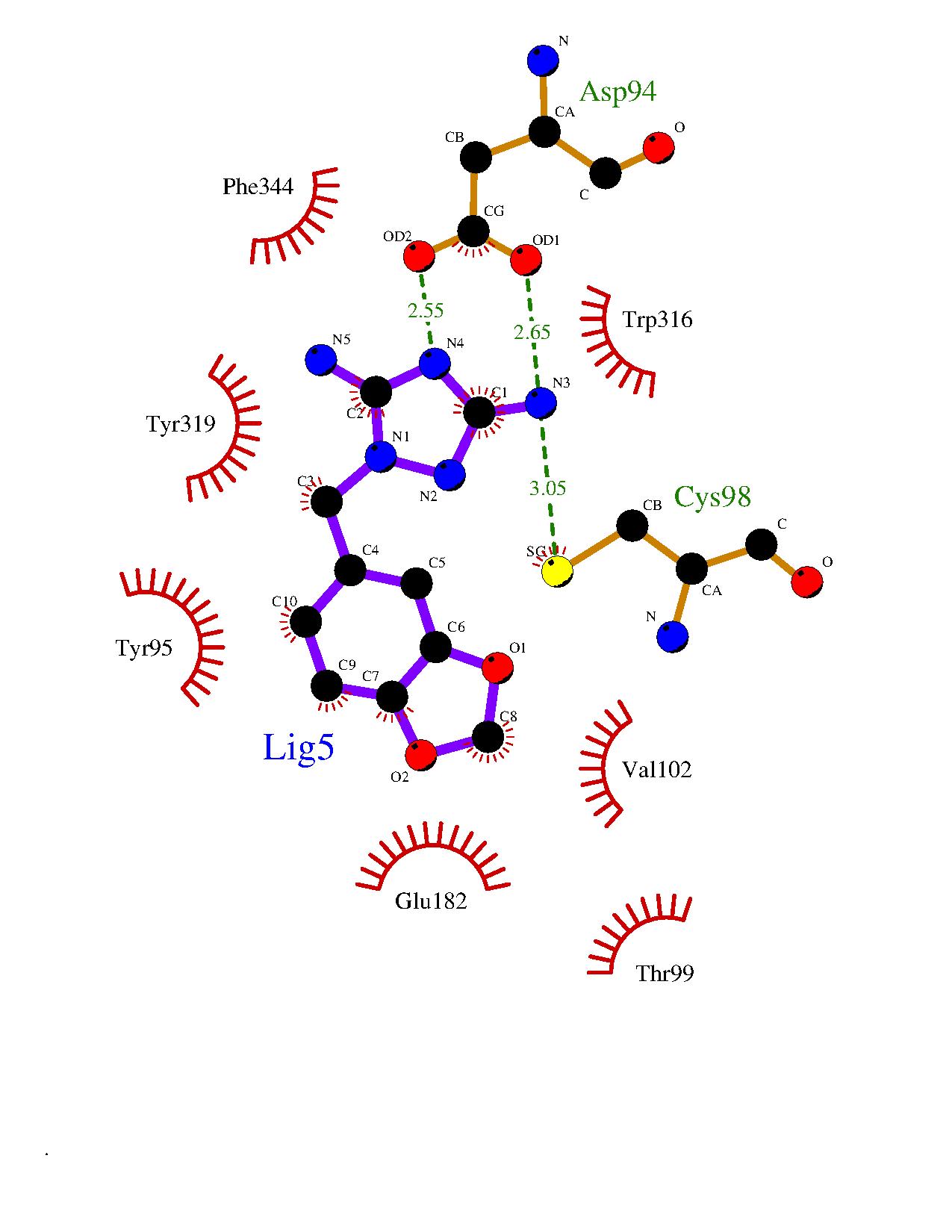


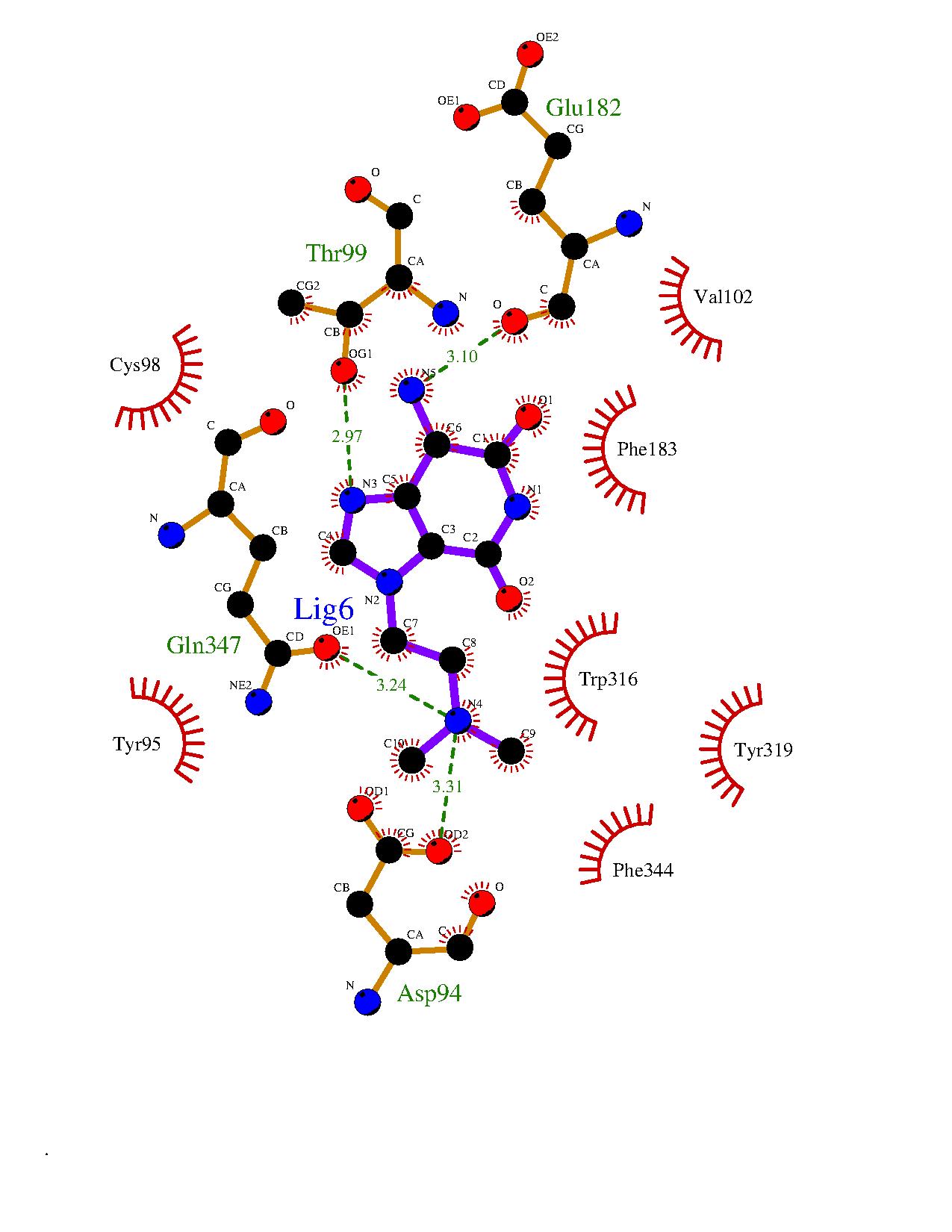


*
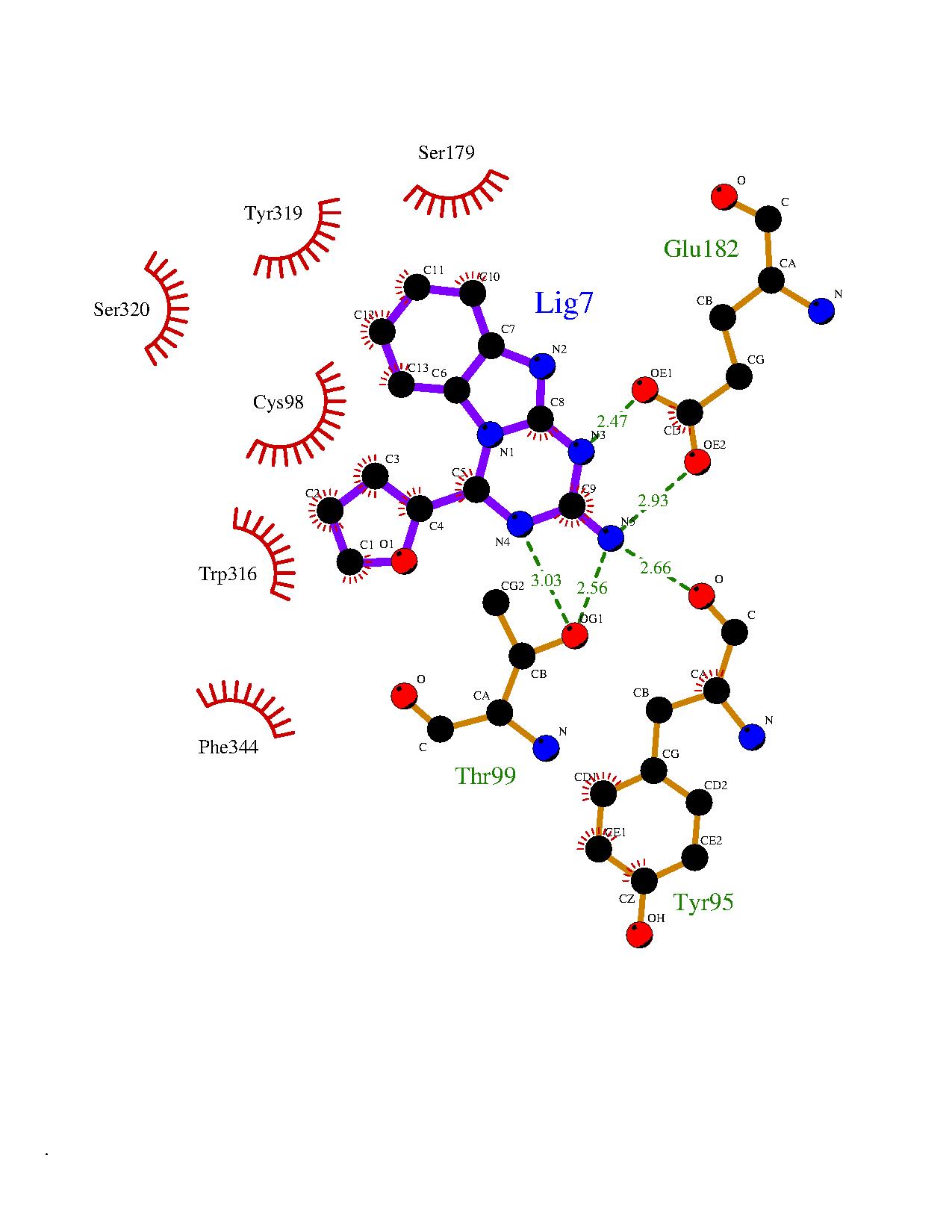
*

*
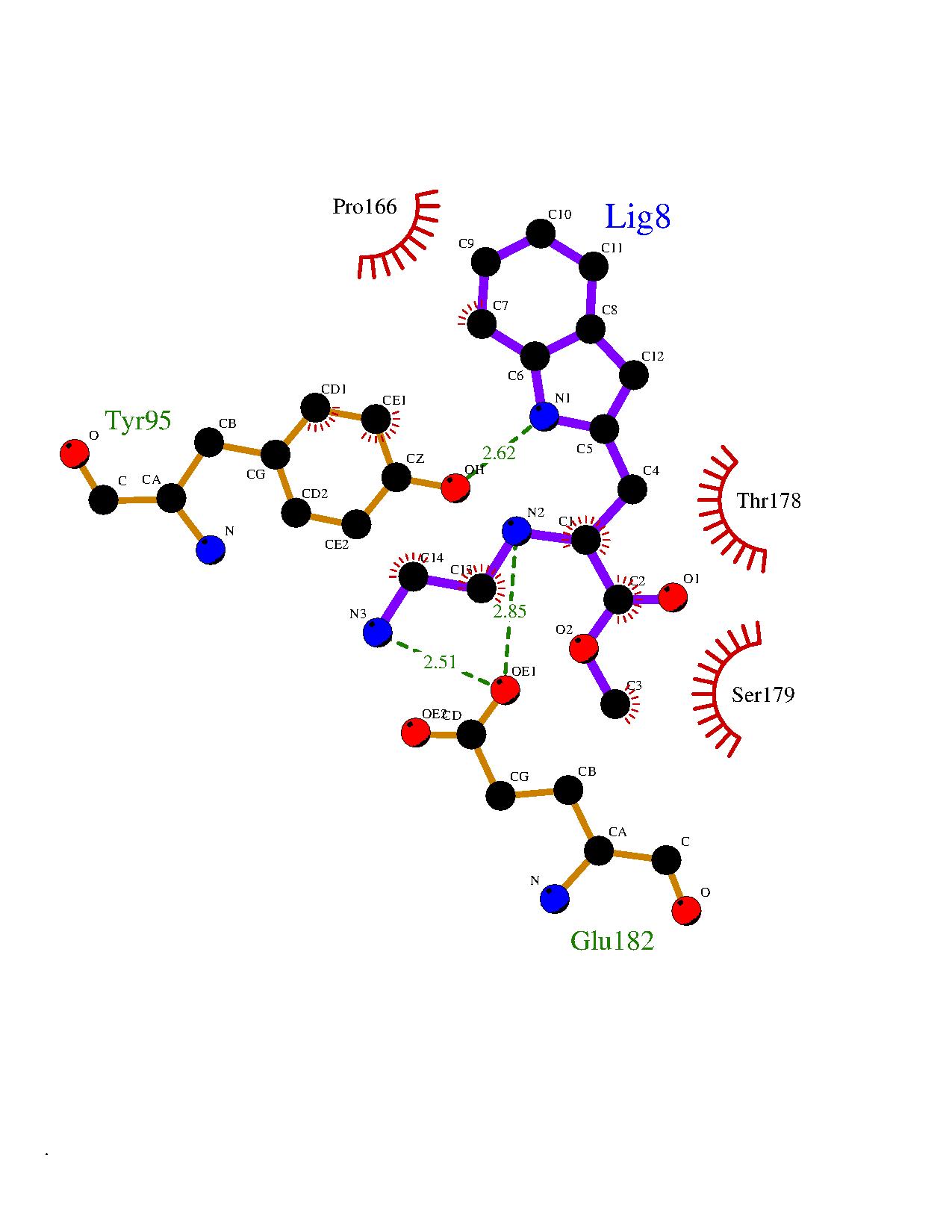
*

*
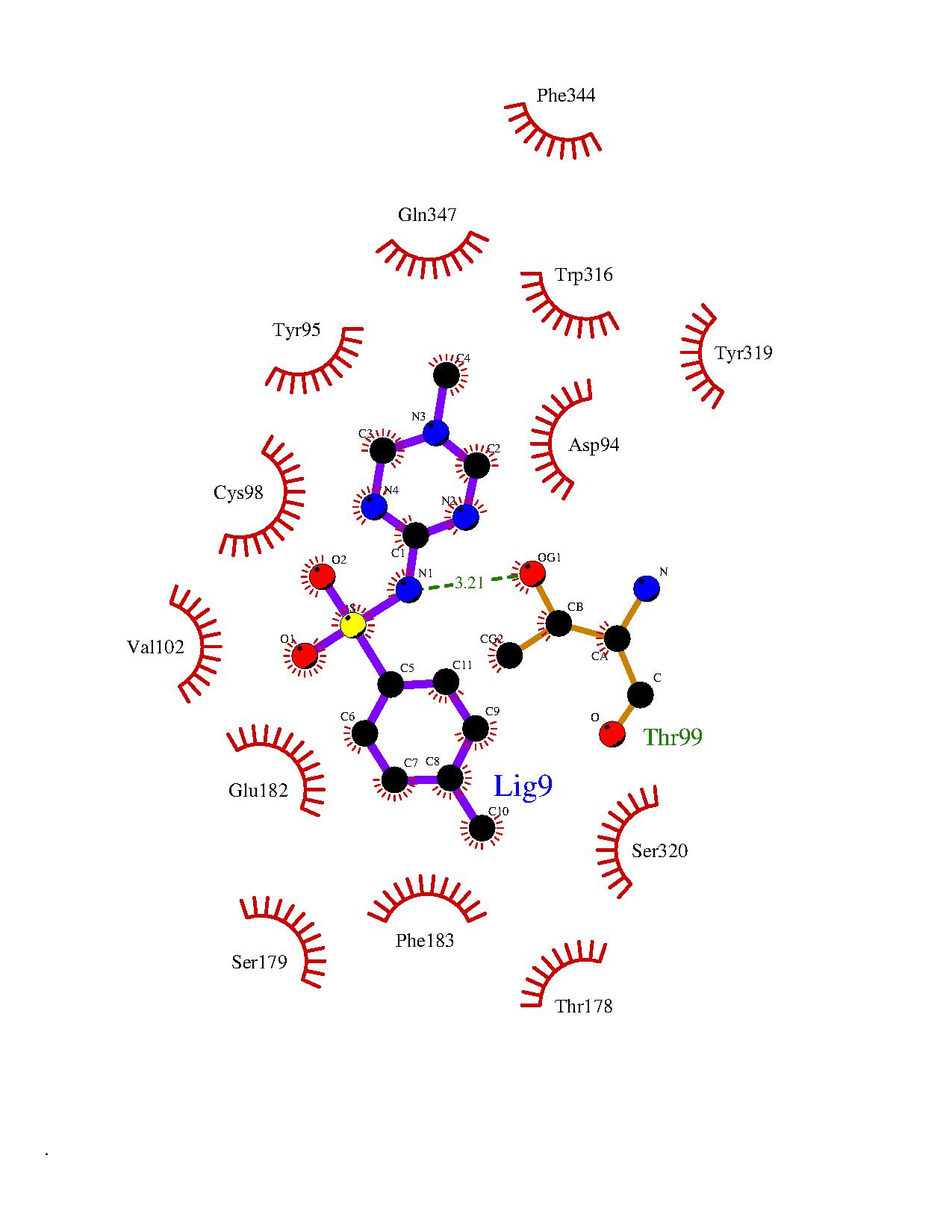
*

*
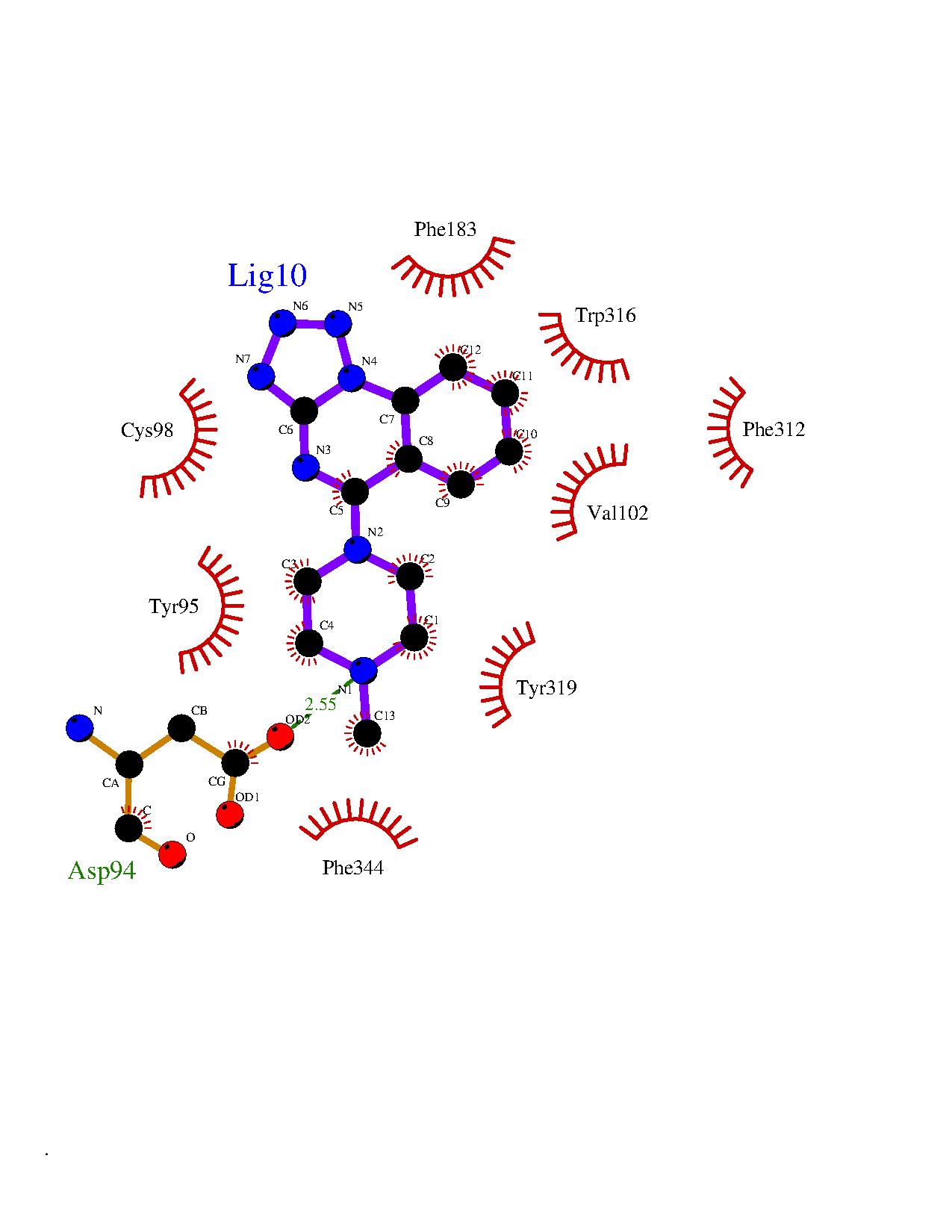
*

*
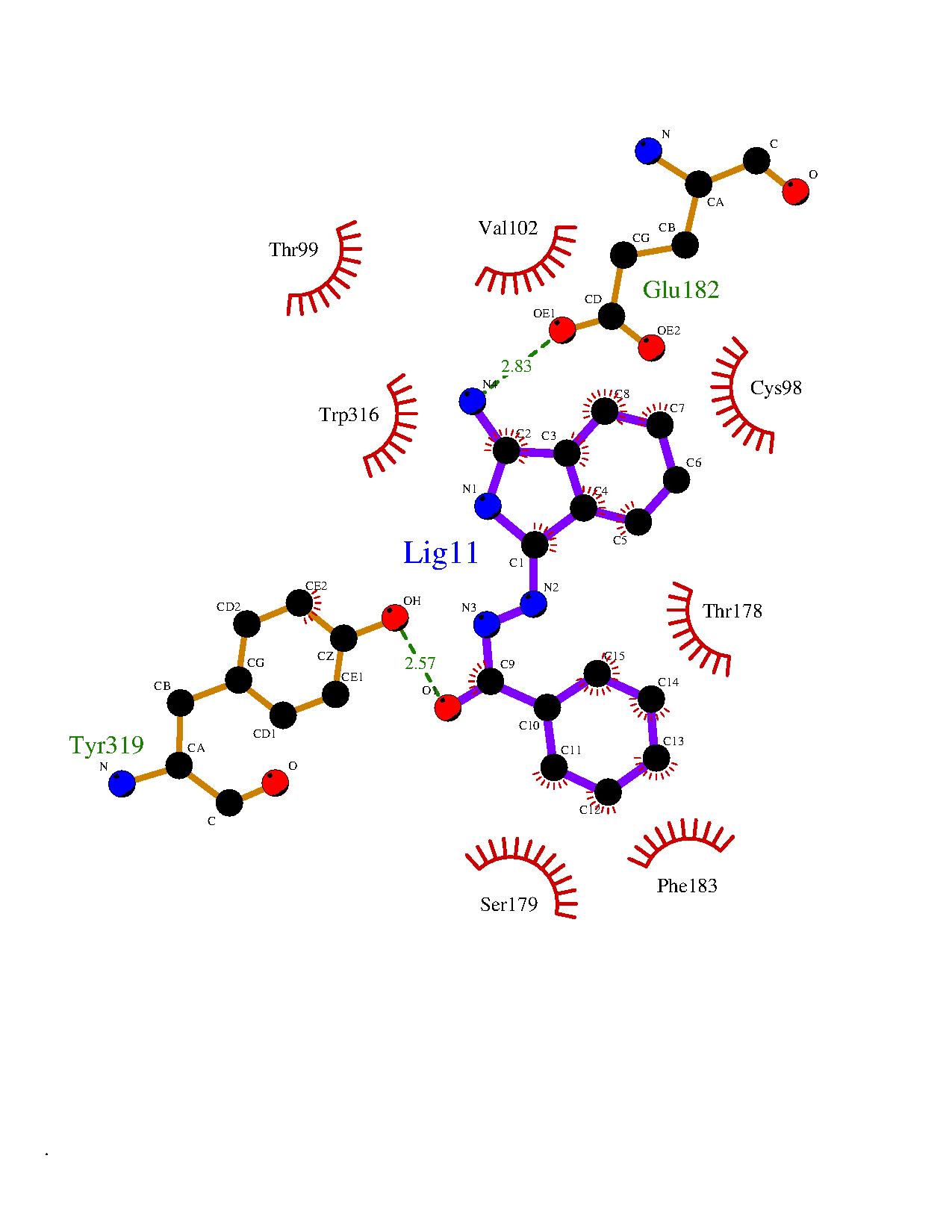
*

SI Figure 2: Two dimensional plots illustrating binding contacts of the candidate hits inside the H4 binding receptor. Hydrogen bonds are represented by a dotted green line, with the distance in Angstrom between heavy atoms marked above. The plots where generated by “LigPlot+” software [Laskowski R A, Swindells M B (2011)] using the 3D structures resulting from the docking procedure (see SI file: “docked candidate hits”).

References:

Laskowski R A, Swindells M B (2011). LigPlot+: multiple ligand-protein interaction diagrams for drug discovery. J. Chem. Inf. Model., 51, 2778-2786.
